# Convergent perturbation of the human domain-resolved interactome by viruses and mutations inducing similar disease phenotypes

**DOI:** 10.1371/journal.pcbi.1006762

**Published:** 2019-02-13

**Authors:** Yangchun Frank Chen, Yu Xia

**Affiliations:** Department of Bioengineering, McGill University, Montreal, Quebec, Canada; University of Chicago, UNITED STATES

## Abstract

An important goal of systems medicine is to study disease in the context of genetic and environmental perturbations to the human interactome network. For diseases with both genetic and infectious contributors, a key postulate is that similar perturbations of the human interactome by either disease mutations or pathogens can have similar disease consequences. This postulate has so far only been tested for a few viral species at the level of whole proteins. Here, we expand the scope of viral species examined, and test this postulate more rigorously at the higher resolution of protein domains. Focusing on diseases with both genetic and viral contributors, we found significant convergent perturbation of the human domain-resolved interactome by endogenous genetic mutations and exogenous viral proteins inducing similar disease phenotypes. Pan-cancer, pan-oncovirus analysis further revealed that domains of human oncoproteins either physically targeted or structurally mimicked by oncoviruses are enriched for cancer driver rather than passenger mutations, suggesting convergent targeting of cancer driver pathways by diverse oncoviruses. Our study provides a framework for high-resolution, network-based comparison of various disease factors, both genetic and environmental, in terms of their impacts on the human interactome.

## Author summary

Cellular function and behaviour are driven by highly coordinated biomolecular interaction networks. A prime example is the protein-protein interaction network, often simply referred to as the “interactome”. Recent advances in systems biology have spawned the view of human disease as a manifestation of genetic and environmental perturbations to the human interactome, a key postulate being that similar perturbation patterns lead to similar disease phenotypes. Here, we took a structural systems biology approach to compare mutation-induced and virus-induced perturbations of the human interactome in diseases with both genetic and viral contributors. Specifically, we constructed a domain-resolved human-virus protein interactome and characterized the distribution of genetic disease mutations with respect to human domains either physically targeted or structurally mimicked by virus. Overall, we found significant convergent perturbation of the human domain-resolved interactome by viruses and mutations inducing similar disease phenotypes. Structure-guided, integrated analysis of host genetic variation and host-pathogen protein interaction data may help elucidate the molecular mechanisms of infection and reveal its connections to genetic diseases such as cancer, autoimmunity, and neurodegeneration. On a broader note, our finding implies that similar perturbations of the human interactome at the domain level can have similar phenotypic consequences, regardless of the source of perturbation.

## Introduction

Cellular function and behaviour are driven by highly coordinated biomolecular interaction networks. A prime example is the protein-protein interaction (PPI) network, also known as the protein “interactome” or interactome for short. A central focus of disease systems biology is to use interactome networks to study genotype-phenotype relationships in complex diseases [1]. The idea of using interactome networks to infer gene function and gene-disease association comes from the well-validated principle of “guilt by association”, which states that physically interacting proteins tend to share similar functions and, by extension, tend to be involved in similar disease processes [1–4]. Recent advances in systems biology have spawned the view of human disease as a manifestation of genetic and environmental perturbations to the human interactome, a key postulate being that similar perturbation patterns lead to similar disease phenotypes [5–8]. A corollary is that, for diseases with both genetic and infectious contributors, similar perturbations of the human interactome by either disease mutations or pathogens can have similar disease consequences. This corollary has been tested for several viral species at the level of whole proteins [9, 10]. For example, Gulbahce *et al*. used yeast two-hybrid screens to map binary interactions between Epstein-Barr virus (EBV) and human papillomavirus (HPV) proteins and human proteins, and transcriptionally profiled human cell lines exogenously expressing HPV oncoproteins E6 and E7 [9]. They found that human genes associated with EBV- and HPV-implicated genetic diseases were often either directly targeted by the virus or transcriptionally regulated by viral targets. This finding led to the idea that oncoviral proteins may preferentially target host proto-oncogenes and tumour suppressors, which was experimentally validated in four families of DNA oncoviruses [10].

Despite insights from these studies on the etiology of virally-implicated genetic diseases, there has yet to be a systematic, structure-based comparison of mutation-induced and pathogen-induced perturbations of the human interactome. A high-resolution, structurally-resolved network biology approach is important for unravelling complex genotype-phenotype relationships, because mutations occurring in different PPI-mediating interfaces on the same protein often have distinct functional impacts and phenotypic consequences [5–8]. In this regard, structural systems biology has proved useful in uncovering evolutionary properties of single- and multi-interface PPI network hubs, systems-level principles governing human-virus interactions, and systems properties of disease variants [6, 11, 12]. For instance, by constructing atomic-resolution human-virus and within-human protein interactomes, Franzosa and Xia discovered that viral proteins tend to target existing endogenous PPI interfaces in the human interactome, rather than creating exogenous interfaces *de novo*, thereby efficiently perturbing multiple endogenous PPIs involved in cell regulation [12]. In a follow-up study, Garamszegi *et al*. expanded the coverage of the human-virus interactome using domain-resolved models of PPIs, and found that viral proteins tend to deploy short linear motifs to bind a variety of human protein domains [13]. The economical and pleiotropic nature of “host domain-viral motif” interactions reflects the efficiency with which viruses rewire the human interactome given limited genomic resources at their disposal. Meanwhile, Wang *et al*. constructed a domain-resolution within-human interactome where protein domains are annotated with disease variant information [6]. They found that mutations occurring in different PPI-mediating domains within the same protein tend to be associated with different disorders (“gene pleiotropy”). By contrast, mutations occurring in the domains of two different but interacting proteins, where the interaction is mediated by said domains, tend to be associated with the same disorder (“locus heterogeneity”). These studies attest to the utility of structural systems biology in the study of infectious and genetic diseases.

Here, we apply structural systems biology to the study of virally-implicated genetic diseases (VIDs), and rigorously test the postulate that endogenous genetic mutations and exogenous viral proteins give rise to similar disease phenotypes by inducing similar perturbations of the human interactome at the level of protein domains. Specifically, we constructed a domain-resolved human-virus protein interactome and characterized the distribution of genetic disease mutations with respect to human domains targeted by virus. Overall, we found that viral proteins and VID mutations induce similar perturbations of the human domain-resolved interactome, for individual viruses with clearly defined VIDs and sufficient numbers of host-virus PPIs (including EBV, HPV and HIV), for oncoviruses, as well as for all viruses combined. We first analyzed the disease associations of host proteins targeted by viral proteins and confirmed that virus-targeted proteins tend to be causally associated with VIDs rather than non-VIDs. We then analyzed the domain-level distribution of disease mutations in virus-targeted proteins and found that virus-targeted domains are significantly enriched for mutations causing VIDs rather than non-VIDs. Using a pooled analysis of all oncoviruses and all oncomutations, we found oncovirus-targeted domains to be significantly enriched for mutations causing cancer rather than other diseases. Furthermore, domains of oncoproteins either physically targeted or structurally mimicked by oncoviruses are significantly enriched for cancer driver mutations rather than passenger mutations, which implies convergent perturbation of cancer driver pathways by diverse oncoviruses. Finally, we also assessed the extent to which viral proteins and VID mutations perturb the same domain-domain interactions (DDIs) in the human interactome. We found that viruses preferentially target DDI partners of domains harbouring VID mutations, regardless of whether the DDI partners themselves are susceptible to known disease mutations. By correlating the equivalent pathogenicity of viral proteins and VID mutations with their convergent perturbation of the human domain-resolved interactome, we provide a framework for high-resolution, network-based comparison of the functional impacts of both genetic and environmental disease factors. On a broader note, our finding implies that similar perturbations of the human interactome at the domain level can have similar phenotypic consequences, regardless of the source of perturbation.

## Results

### Disease-annotated, domain-resolved human-virus protein interaction network

We first acquired human-endogenous and human-virus binary PPI data from IntAct, HPIDB 3.0, and the HIV-1 Human Interaction Database [14–18]. Only PPIs supported by at least one PubMed ID were included in the whole-protein resolution human-virus interactome, which consists of 173830 PPIs between 15995 human proteins, and 28531 PPIs between 7761 human proteins and 624 viral proteins. 7211 human proteins participate in both endogenous and exogenous PPIs. To build homology models of PPIs, we collected high-confidence domain-domain interaction (DDI) and domain-motif interaction (DMI) templates derived from 3D structures of protein complexes in the Protein Data Bank, and scanned protein sequences for the occurrence of Pfam domains and domain-binding linear motifs [19–23]. Structural models were assigned to each PPI by extracting all DDIs and DMIs possibly mediating the PPI. The resulting domain-resolved human-virus structural interaction network (hvSIN) consists of 61041 PPIs between 11596 human proteins, and 4654 PPIs between 1590 human proteins and 405 viral proteins. 1517 human proteins participate in both endogenous and exogenous portions of hvSIN.

We then obtained manually-curated disease variant data from UniProtKB and ClinVar [24, 25], selecting missense variants located inside Pfam domains for our analyses. Overall, 19047 mutations associated with 5383 diseases were mapped to 3585 domains of 2622 proteins. 14720 mutations associated with 4185 diseases were mapped to 2642 domains of 1864 human proteins in hvSIN. Table 1 lists the number of mutations by the type of domain in which they occur. Incidentally, 1272 domains of 957 human proteins in hvSIN are susceptible to disease mutations, but lack interacting domains or motifs. 850 of these 1272 domains harbour a total of 4154 mutations associated with 1381 diseases that are not accounted for by mutations occurring in PPI-mediating domains in hvSIN. Because the completeness of a domain’s PPI profile depends largely on the interactome search space and availability of 3D structures of protein complexes, and domains often have important biological functions besides mediating PPIs (*e*.*g*. enzymatic or nucleotide-binding activity), we included all domains of virus-targeted host proteins in a comprehensive analysis of the domain-level distribution of disease mutations.

**Table 1:** Number of disease mutations mapped to human protein domains in the human-virus structural interaction network (hvSIN).

	Proteins	Domains	Disease mutations	Diseases
All disease proteins in hvSIN	1864	2642	14720	4185
Disease proteins containing exclusively endogenously-interacting domains	924	1147	7073	2281
Disease proteins containing exclusively exogenously-interacting domains	9	9	19	15
Disease proteins containing overlapping endogenous-exogenous domains	200	214	1300	583
Disease proteins containing domains without annotated interacting domains or motifs	957	1272	6328	2224

### Virus-targeted host domains are enriched for virally-implicated disease mutations

**Fig 1:**
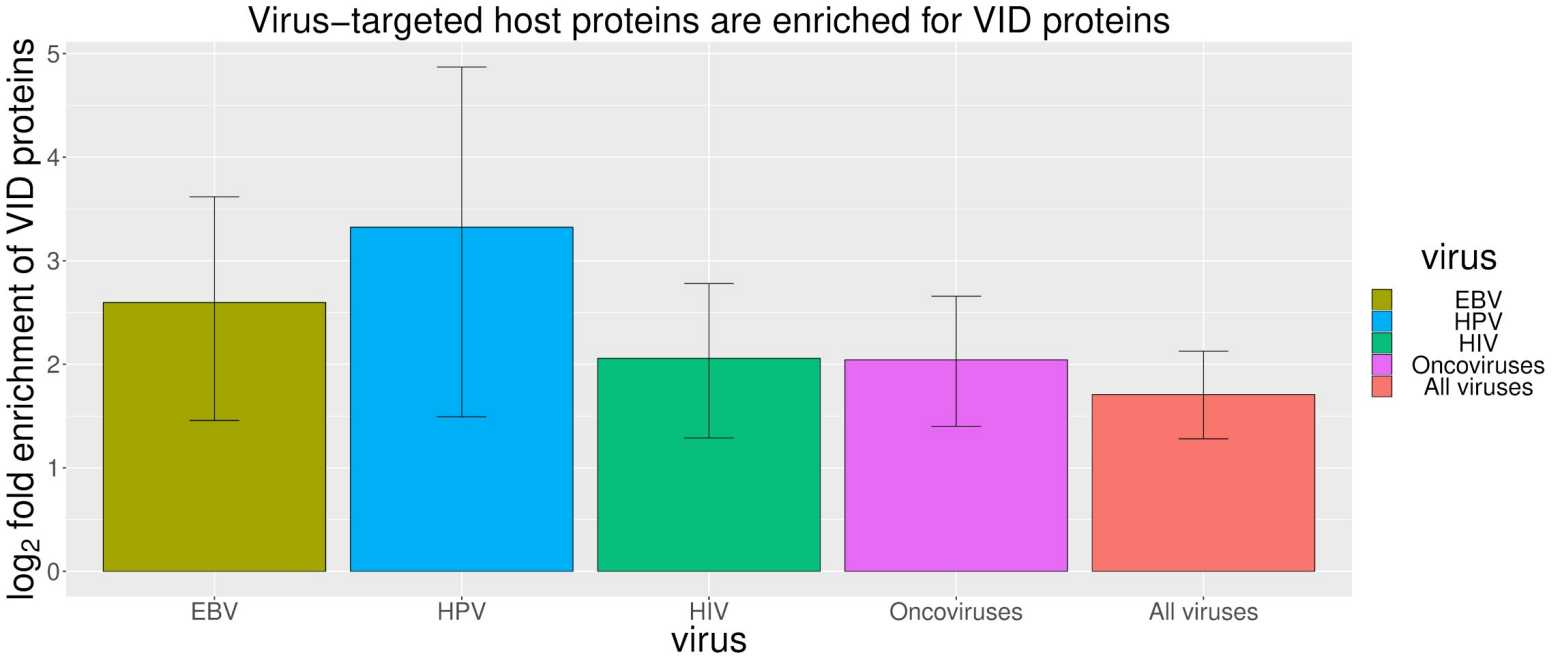
Virus-targeted host proteins tend to be causally associated with virally-implicated diseases (VIDs). “VID proteins” have at least one missense variant that is causally associated with a VID, whereas all missense variants of “non-VID proteins” are exclusively associated with non-VIDs. Literature-curated, virus-specific diseases for EBV, HPV and HIV are listed in S1 Table. For pooled analysis of oncoviruses, VIDs include all types of cancer (Methods). For pooled analysis of all viruses, VIDs include all proliferative and immunological diseases (Methods). Error bars represent 95% confidence intervals.

**Fig 2:**
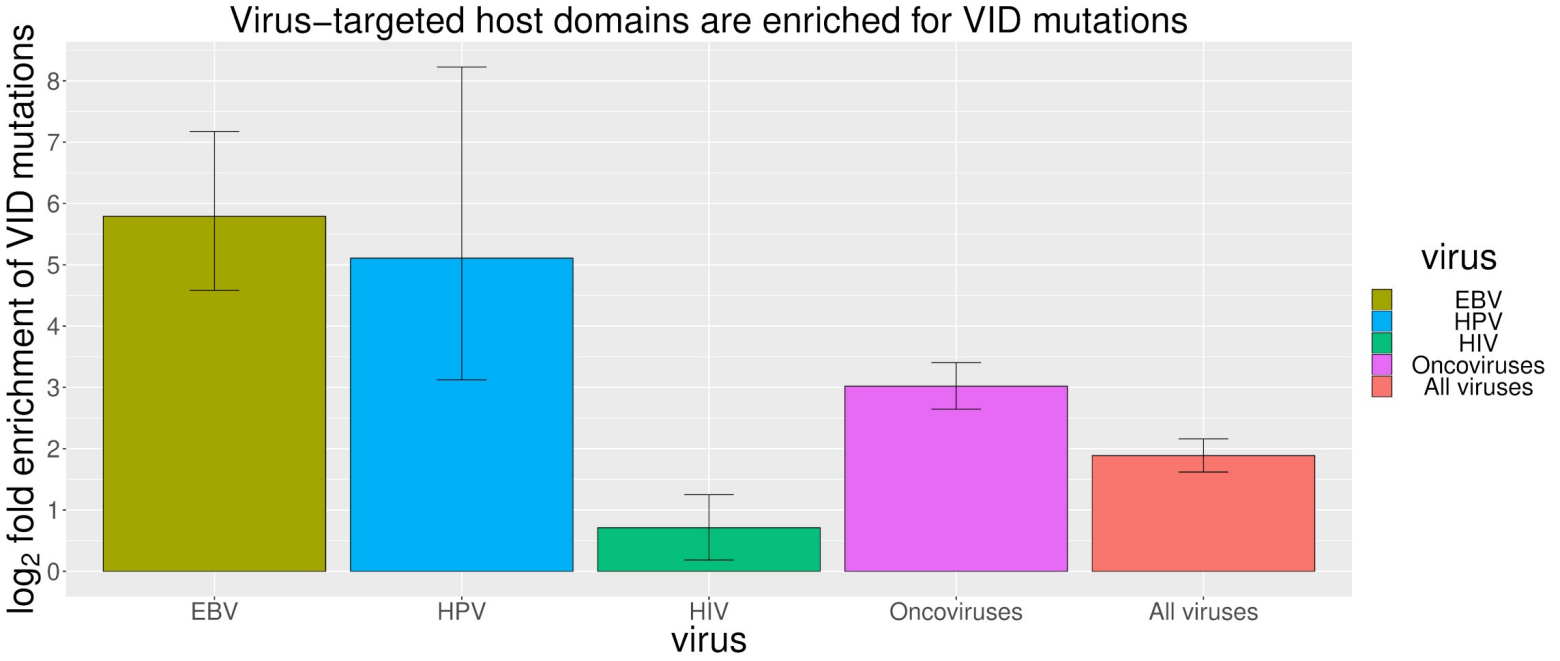
Virus-targeted host domains tend to harbour mutations causally associated with virally-implicated diseases (VIDs). “VID mutations” are causally associated with at least one VID, whereas “non-VID mutations” are exclusively associated with non-VIDs. Error bars represent 95% confidence intervals.

To relate the equivalent pathogenicity of viral proteins and VID mutations to their equivalent perturbation of the host interactome, we first characterized the mutational landscape of human proteins targeted by EBV, HPV and HIV, three viruses with clearly defined VIDs and sufficient numbers of host-virus PPIs. Since most oncoviruses are causally implicated in only a few site-specific malignancies (*e*.*g*. HBV/HCV in hepatocellular carcinoma, KSHV in Kaposi’s sarcoma, and HTLV in adult T-cell lymphoma), and various types of cancer share common molecular hallmarks [26, 27], to increase the statistical power of our analysis and establish whether a general equivalence exists between endogenous and exogenous perturbagens of oncogenic pathways, we also performed a pooled analysis of host proteins targeted by diverse oncoviruses, by considering all types of cancer as interchangeable diseases, all oncomutations as interchangeable endogenous perturbagens, and all oncoviral proteins as interchangeable exogenous perturbagens. We found that for EBV, HIV, HPV and a broad spectrum of oncoviruses, virus-targeted host proteins tend to be causally associated with VIDs (Fig 1), and virus-targeted host domains tend to harbour mutations causally associated with VIDs (Fig 2). We discuss our findings for each type of virus below. A full list of VIDs and disease-associated proteins for EBV, HPV and HIV can be found in S1 Table.

#### EBV

**Fig 3:**
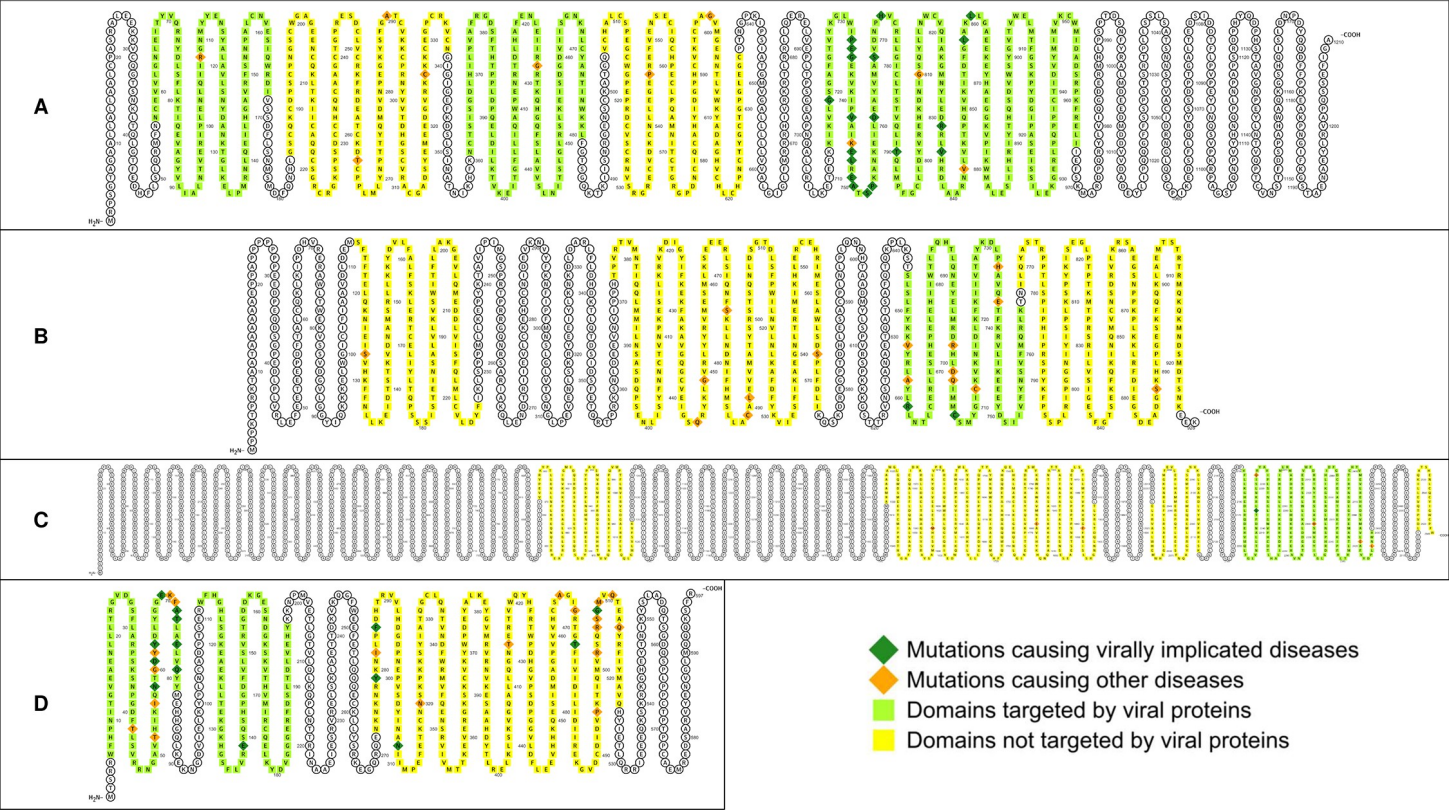
Exclusive localization or enrichment of VID mutations in virus-targeted domains. (A) Exclusive localization of mutations causing lung cancer, an EBV-implicated disease, in EBV-targeted tyrosine kinase domain of *EGFR* protein. (B) Exclusive localization of mutations causing vulvar cancer and lung cancer, both HPV-implicated diseases, in HPV-targeted B domain of *RB* protein. (C) Exclusive localization of mutations causing cervical cancer, an HIV-implicated disease, in HIV-targeted PI3-kinase domain of *MTOR* protein, while mutations causing other diseases such as focal cortical dysplasia and Smith-Kingsmore syndrome are evenly distributed among all domains of *MTOR*. (D) Moderate enrichment of oncomutations in KSHV-targeted SH2 domain of *PTPN11* protein, compared to mutations causing Noonan syndrome. Most of the oncomutations cause juvenile myelomonocytic leukemia, a disease although not caused by KSHV, is mimicked clinically and morphologically by other human herpesvirus infections, including EBV, CMV and HHV-6. VID mutations are shown as dark green diamonds. Non-VID mutations are shown as orange diamonds. Amino acid residues in virus-targeted domains are shown as light green squares. Residues in domains not targeted by virus are shown as yellow squares.

EBV is involved in lymphomas of the B, T, and NK-cell lineages as well as in adenocarcinomas of epithelial cells [28–32]. EBV hijacks cellular signaling processes by encoding viral homologues of cellular proteins that play key roles in apoptosis and proliferation. Examples include *EBNA2* (mimics Notch signaling), *LMP1* (mimics CD40 receptor signaling), *LMP2* (mimics IgG receptor signaling), *BALF1* and *BHRF1* (homologues of cellular *Bcl-2*), and *BCRF1* (homologue of cellular *IL-10*) [27]. All EBV homologues share at least one PPI partner with their cellular counterparts. Overall, EBV targets 11/99 (11.1%) host proteins associated with EBV diseases, and 51/2523 (2%) host proteins associated with non-EBV diseases, *i*.*e*. EBV tends to directly target host proteins causally associated with EBV-implicated diseases (Fisher’s exact test, two-tailed P = 1 × 10^−5^) (Fig 1). Analysis of the domain-level distribution of disease mutations found that 35/43 (81.4%) EBV-disease mutations and 62/856 (7.2%) non-EBV disease mutations occur in EBV-targeted domains, suggesting that EBV-targeted domains are significantly enriched for EBV-disease mutations (Fisher’s exact test, two-tailed P < 2.2 × 10^−16^) (Fig 2). Fig 3A shows the exclusive localization of mutations causing lung cancer, an EBV-implicated disease, in EBV-targeted tyrosine kinase domain (PF07714) of *EGFR* protein, while mutations causing other diseases such as brain cancer are evenly distributed among all domains of *EGFR*.

#### HPV

High-risk human papillomaviruses (HPV16, 18, 31, 33, 35, 39, 45, 51, 52, 56, 58, 59, 66, 68), as defined by the Centers for Disease Control and Prevention (CDC) and International Agency for Research on Cancer (IARC), are established etiological agents for cervical, oropharyngeal and anogenital cancers [33–35]. Several studies have also reported an association between HPV and cancers of the bladder [36], breast [37], lung [38], and prostate [39]. Overall, HPV targets 5/79 (6.3%) host proteins associated with HPV diseases, and 17/2543 (0.7%) host proteins associated with non-HPV diseases, *i*.*e*. HPV tends to directly target host proteins causally associated with HPV-implicated diseases (Fisher’s exact test, two-tailed P = 3 × 10^−4^) (Fig 1). Analysis of the domain-level distribution of disease mutations found that 117/119 (98.3%) HPV-disease mutations and 94/150 (62.7%) non-HPV disease mutations occur in HPV-targeted domains, suggesting that HPV-targeted domains are significantly enriched for HPV-disease mutations (Fisher’s exact test, two-tailed P = 2 × 10^−14^) (Fig 2). Fig 3B shows the exclusive localization of mutations causing vulvar cancer and lung cancer, both HPV-implicated diseases, in HPV-targeted B domain (PF01857) of *RB* protein, while mutations causing other diseases such as retinoblastoma are evenly distributed among all domains of *RB*.

#### HIV

HIV substantially raises the risk of Kaposi’s sarcoma, non-Hodgkin’s lymphoma and cervical cancer [40], as well as cancers of the anus, liver, lung, oropharynx and testes [41]. Although HIV-encoded accessory proteins such as *Tat* and *Nef* have demonstrated oncogenic properties on their own [42–44], HIV-associated cancers are mostly attributed to opportunistic infections with oncoviruses such as KSHV, EBV, HPV, and Hepatitis B/C virus. In addition, other HIV-associated complications such as cardiomyopathy and neurocognitive disorders have become increasingly common in the post-HAART era [45–50]. Overall, HIV targets 23/132 (17.4%) host proteins associated with HIV diseases, and 120/2490 (4.8%) host proteins associated with non-HIV diseases, *i*.*e*. HIV tends to directly target host proteins causally associated with HIV-implicated diseases (Fisher’s exact test, two-tailed P = 3 × 10^−7^) (Fig 1). Analysis of the domain-level distribution of disease mutations found that 103/158 (65.2%) HIV-disease mutations and 479/898 (53.3%) non-HIV disease mutations occur in HIV-targeted domains, suggesting that HIV-targeted domains are significantly enriched for HIV-disease mutations (Fisher’s exact test, two-tailed P = 7 × 10^−3^) (Fig 2). Fig 3C shows the exclusive localization of mutations causing cervical cancer, an HIV-implicated disease, in HIV-targeted PI3-kinase domain (PF00454) of *MTOR* protein, while mutations causing other diseases such as focal cortical dysplasia and Smith-Kingsmore syndrome are evenly distributed among all domains of *MTOR*. In addition to offering general insights on human-HIV interaction, our domain-resolved PPI models also provide useful information about specific HIV proteins. For instance, our model for the interaction between human *Akt1* and HIV *Nef* involves the protein kinase domain (PF00069) of *Akt1* and a region of *Nef* matching three overlapping motifs: MOD_NEK2_1 (residues 100–105), DOC_MAPK_gen_1 (residues 105–112) and DOC_MAPK_MEF2A_6 (residues 105–114). Notably, our predicted *Akt1*-binding region of *Nef* (residues 100–114) is consistent with the experimentally determined *Akt1*-binding region of *Nef* (residues 55–210) [51]. hvSIN also reveals a previously unreported similarity between the host interaction profiles of HIV *Nef* and the EBV oncoprotein *LMP2*, in that both can bind the SH2 domain (PF00017) of *Src* family kinases (*Lck*, *Lyn*, *Src*) and *Syk* family kinases (*Syk*, *ZAP70*), as well as the WW domain (PF00397) of the *Nedd4* family of E3 ubiquitin ligases (*Itch*, *Nedd4*), possibly revealing disease modules perturbed in common by HIV and EBV in AIDS-related lymphoma [52, 53].

#### Oncoviruses

Oncoviruses contribute to 12% of human cancers worldwide and can activate in a cancer cell the same molecular hallmarks shared among cancers of non-viral origin [27, 54]. In fact, some of the most potent oncogenes were first discovered in retroviruses [55]. Oncoviruses in hvSIN include human herpesviruses (HHV-4/EBV, HHV-5/CMV, HHV-8/KSHV), high-risk HPVs, human polyomaviruses (BKV, JCV, MCV), hepatitis B and C viruses, human T cell lymphotropic virus (HTLV) and oncogenic retroviruses. Some oncoviruses, although not directly infectious to human, are tumorigenic in other species, can transform human cells *in vitro*, and serve as models for studying viral oncogenesis in human (*e*.*g*. murid herpesvirus 4) [56, 57]. Despite HIV being classified by IARC as a Group 1 carcinogen and the *in vitro* oncogenicity of HIV-encoded accessory proteins, we excluded it from the pooled analysis of oncoviruses, because there is insufficient data on HIV prevalence and cancer incidence among HIV-infected individuals to accurately assess the independent contribution of HIV to infection-attributable cancers [58]. Pooled analysis of all oncovirus-targeted host proteins found that oncoviruses target 34/194 (17.5%) oncoproteins and 119/2428 (4.9%) proteins associated with non-cancer diseases, *i*.*e*. oncoviruses tend to directly target oncoproteins (Fisher’s exact test, two-tailed P = 1 × 10^−9^) (Fig 1). Analysis of the domain-level distribution of disease mutations found that 314/413 (76%) oncomutations and 371/1322 (28.1%) other disease mutations occur in oncovirus-targeted domains (OVTDs), *i*.*e*. the odds of finding cancer-causing over other disease-causing mutations in OVTDs is 8 times as high as that in non-OVTDs (Fisher’s exact test, two-tailed P < 2.2 × 10^−16^) (Fig 2). Fig 3D shows a moderate enrichment of oncomutations in KSHV-targeted SH2 domain (PF00017) of *PTPN11* protein, compared to mutations causing Noonan syndrome. Most of the oncomutations cause juvenile myelomonocytic leukemia, a disease although not caused by KSHV, is mimicked clinically by other human herpesvirus infections, including EBV, CMV and HHV-6 [59, 60]. Finally, we also assessed the mutational landscape of 107 oncovirus-targeted pleiotropic proteins that are susceptible to both oncomutations and other disease mutations. Overall, 88/113 (77.9%) oncomutations and 110/179 (61.5%) other disease mutations were mapped to the OVTDs of these pleiotropic proteins, suggesting that enrichment of oncomutations in OVTDs holds even at the level of individual proteins involved in both cancer and other diseases (Fisher’s exact test, two-tailed P = 4 × 10^−3^).

#### Viruses in proliferative and immunological diseases

**Fig 4:**
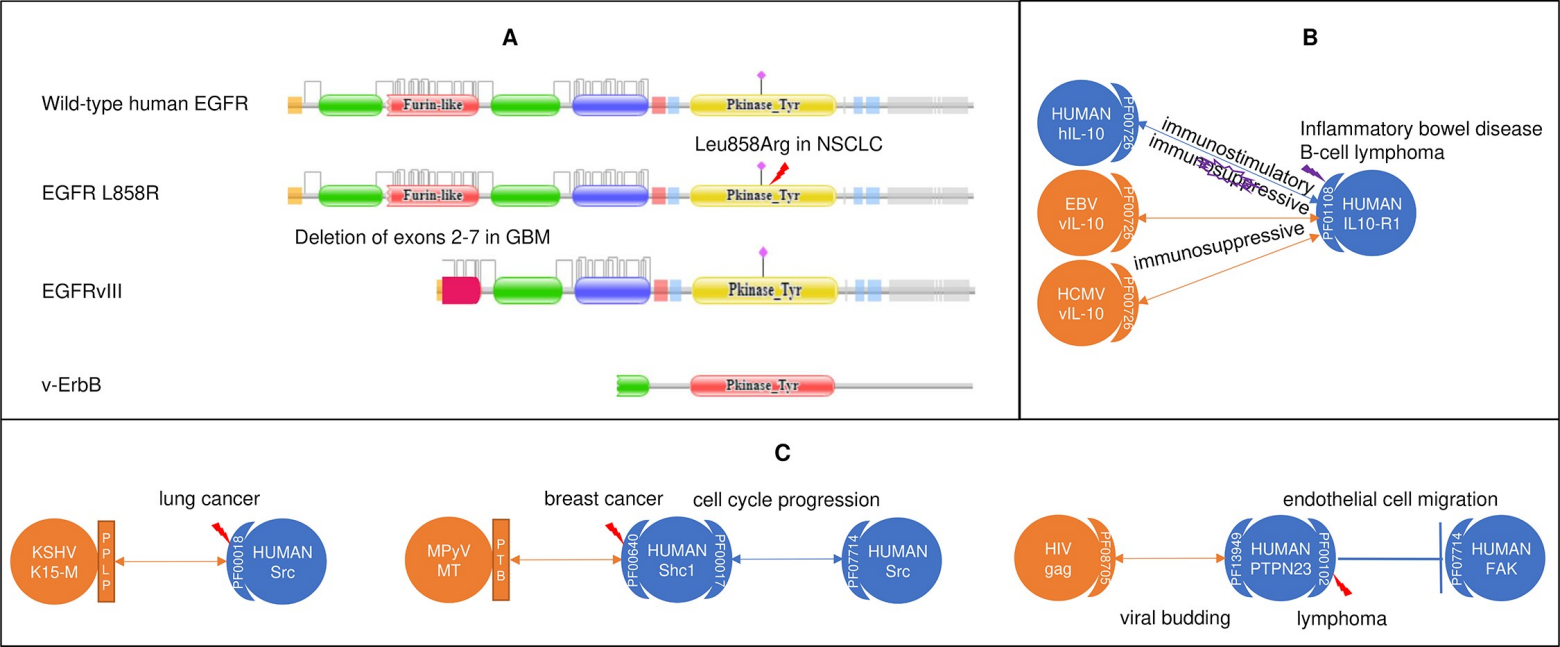
Viral and mutational perturbations of host domains are mechanistically similar. (A) Viruses encode homologues of human proteins to mimic mutations in oncoproteins that cause uncontrolled cell proliferation. Top: EGFRvIII deletion mutation, frequently detected in glioblastoma multiforme (GBM) patients, and *v-ErbB*, encoded by avian leukosis virus, both lack the *EGFR* ligand-binding domain. Meanwhile, an L858R missense mutation in the *EGFR* kinase domain is frequently found in non-small-cell lung cancer (NSCLC). These alterations lead to conformational changes that result in ligand-independent, constitutive kinase activity [61, 62]. (B) Viruses encode homologues of human proteins to antagonize mutations in cytokine receptors that cause hypersensitivity. Human *IL-10* functions both as an immunosuppressant in the inhibition of proinflammatory cytokines, and as an immunostimulant in the induction of MHC II expression on B cells. Mutations in the *IL10*-binding domain of *IL-10R1* abrogate *hIL10*-induced phosphorylation, leading to loss of immunosuppression and inflammatory bowel disease [63]. In contrast, viral *IL-10* homologues encoded by Epstein-Barr virus (EBV) and human cytomegalovirus (HCMV) retain and amplify the immunosuppressive properties of *hIL-10*, thus facilitating viral persistence after lytic infection [64]. *ebvIL-10* selectively retains only the immunosuppressive properties of *hIL-10*. *cmvIL-10* binds with greater affinity to *IL-10R1* than *hIL-10*, while co-opting other *IL10*-associated pathways to amplify the immunosuppressive properties of *hIL-10*. Interestingly, transgenic expression of v*IL-10* has been tested in animal models as an immunosuppressant option for transplant recipients [65]. In addition, abnormal expression levels of *IL-10*, *IL10-R1* and *IL10-R2* has been suggested as a mechanism for diffuse large B-cell lymphoma, a disease with clear EBV involvement [66]. (C) Viruses abuse peptide motifs to modulate host signaling pathways, potentially mimicking the effects of disease-causing mutations. Left: Kaposi’s sarcoma-associated herpesvirus (KSHV) protein *K15-M* uses a “PPLP” motif to bind the SH3 domain (PF00018) of *Src* [67], which possibly induces conformational opening of the *Src* kinase domain, thereby mimicking activating mutations such as Y527F [68]. Interestingly, a W121C mutation in the KSHV-targeted SH3 domain of *Src* has been identified in lung cancer [69]. Middle: Murine polyomavirus (MPyV) Middle T antigen (*MT*) uses a tyrosine-phosphorylated motif to recruit host *Shc1*, thereby promoting cell cycle progression [70]. Interestingly, a R175Q mutation in the MPyV-targeted PTB domain (PF00640) of *Shc1* has been found to regulate tumorigenesis in mouse models of breast cancer [71]. Right: HIV protein *gag* uses the late-budding domain to sequester host *PTPN23* and facilitate viral budding [72]. The phosphatase domain (PF00102) of *PTPN23* regulates cell migration via dephosphorylation of *FAK* and is often mutated in cancer and developmental disorders [73, 74].

All viruses have evolved sophisticated mechanisms to subvert host transcriptional and signaling machineries for replication and persistence. Viruses are known to encode homologues of cellular proteins to mimic mutant oncoproteins (Fig 4A) or antagonize mutant cytokine receptors (Fig 4B). Viruses have also been shown to abuse peptide motifs to modulate host signaling pathways, potentially mimicking the effects of disease-causing mutations (Fig 4C). We suspect that viruses and mutations causing proliferative and immunological diseases (PIDs) target similar human domains involved in cell cycle progression, apoptosis, DNA repair and immune homeostasis. Proliferative diseases include various neoplasms, both benign and malignant. Examples include lung cancer (Fig 3A), vulvar and lung cancer (Fig 3B), cervical cancer (Fig 3C), juvenile myelomonocytic leukemia (Fig 3D), glioblastoma multiforme and non-small-cell lung cancer (Fig 4A), lung cancer, breast cancer and lymphoma (Fig 4C). Immunological diseases include autoimmune diseases, hypersensitivity, and immunodeficiency disorders. One example of an immunological disease, inflammatory bowel disease (IBD), is given in Fig 4B, where we show convergent perturbation of the *IL10*-binding domain of *IL-10R1* by both viral homologues of *IL-10* and IBD mutations.

To establish whether a general equivalence exists between endogenous and exogenous perturbagens of pathways associated with PIDs, we performed a pooled analysis of all virus-targeted host proteins by considering all PIDs as a unique category of diseases with both genetic and viral contributors, all PID mutations as interchangeable endogenous perturbagens, and all viral proteins as interchangeable exogenous perturbagens. We found that overall, viruses tend to target host proteins associated with PIDs (85/338, 25.1%) rather than non-PIDs (213/2284, 9.3%) (Fisher’s exact test odds ratio = 3.3, two-tailed P = 1 × 10^−14^) (Fig 1), and virus-targeted domains are enriched for mutations causing PIDs (525/737, 71.2%) rather than non-PIDs (803/2003, 40%) (Fisher’s exact test odds ratio = 3.7, two-tailed P < 2.2 × 10^−16^) (Fig 2). Since the equivalence between oncoviruses and oncomutations has already been established in the previous section, we excluded proliferative diseases from consideration and further tested the equivalence between viral proteins and mutations in causing immunological diseases. Again, we found that viruses tend to target host proteins associated with immunological diseases (31/151, 20.5%) rather than other diseases (267/2471, 10.8%) (Fisher’s exact test odds ratio = 2.1, two-tailed P = 8 × 10^−4^), and virus-targeted domains are enriched for mutations causing immunological diseases (101/179, 56.4%) rather than other diseases (1227/2561, 47.9%) (Fisher’s exact test odds ratio = 1.4, two-tailed P = 0.03). Finally, we tested the equivalence between viral proteins and mutations in causing proliferative, but not immunological diseases. Overall, viruses tend to target host proteins associated with proliferative diseases (56/199, 28.1%) rather than other diseases (242/2423, 10%) (Fisher’s exact test odds ratio = 3.5, two-tailed P = 8 × 10^−12^), and virus-targeted domains are enriched for mutations causing proliferative diseases (431/571, 75.5%) rather than other diseases (897/2169, 41.4%) (Fisher’s exact test odds ratio = 4.4, two-tailed P < 2.2 × 10^−16^).

### Oncovirus-targeted host domains are enriched for cancer driver mutations

A main challenge in cancer research is to distinguish mutations which confer clonal growth advantage (*i*.*e*. drivers), from mutations that do not cause clonal expansion (*i*.*e*. passengers) [75]. Large-scale cancer genome sequencing projects have enabled systematic identification of cancer driver proteins and mutations [76]. Rozenblatt-Rosen *et al*. previously constructed an oncovirus-human interactome and demonstrated, at the whole-protein level, comparability between oncoviral perturbation and conventional functional genomics approaches to cancer gene discovery [10]. However, by representing proteins and PPIs as generic nodes and edges, their approach is not sensitive enough to distinguish driver mutations from passenger mutations occurring in the same oncoprotein. As we demonstrated earlier in the case of pleiotropic oncoproteins, the oncogenicity or “driver-ness” of a mutation is often correlated with its occurrence in oncovirus-targeted domains (OVTDs).

**Fig 5:**
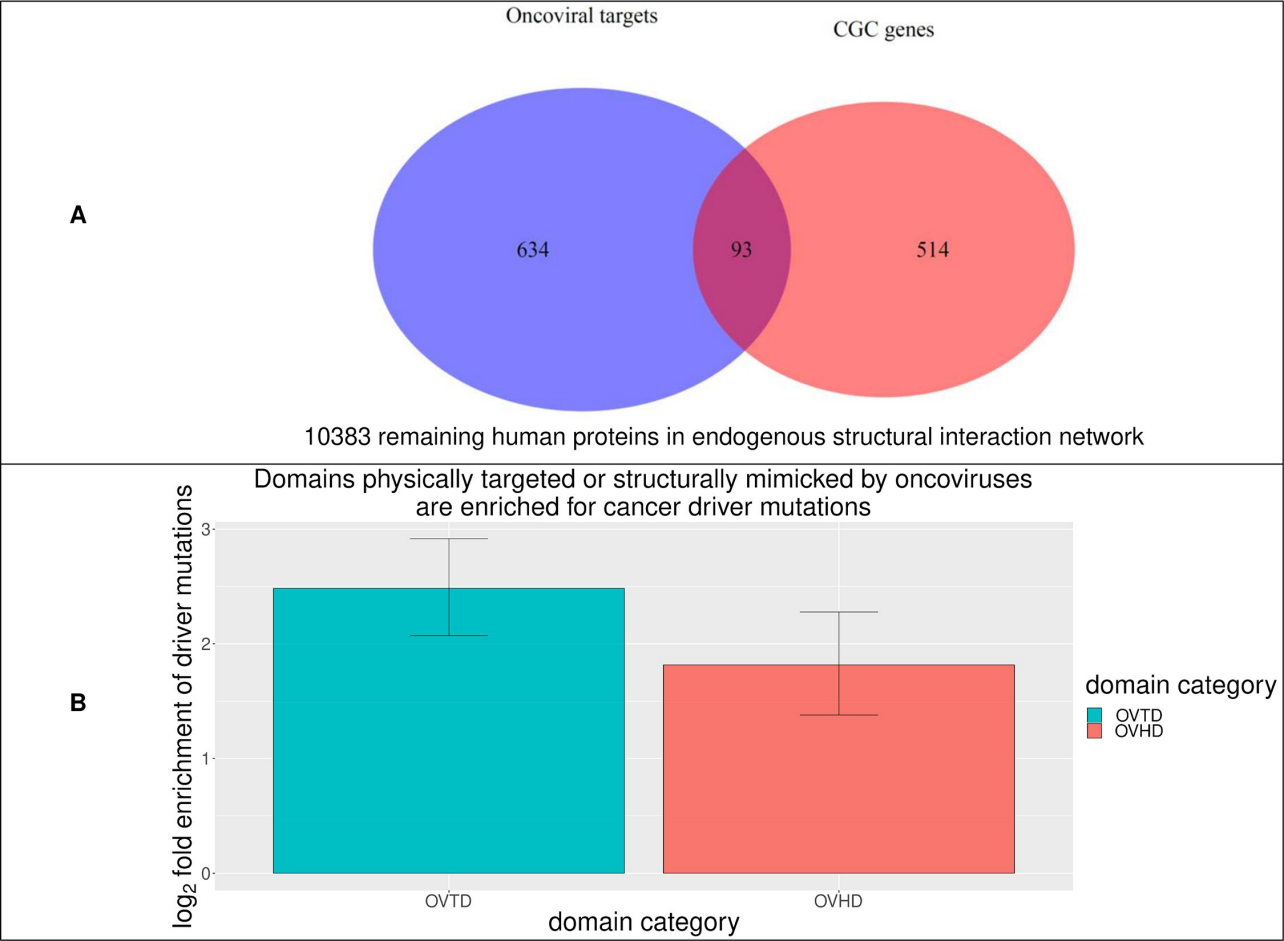
Oncovirus-targeted proteins are enriched for driver proteins, and oncovirus-targeted or mimicked domains are enriched for driver mutations. (A) There is a 3-fold enrichment of Cancer Gene Census proteins in oncovirus-targeted proteins. (B) There are 5-fold and 3-fold enrichments of driver mutations in oncovirus-targeted domains (OVTDs) and oncoviral homology domains (OVHDs), respectively.

**Fig 6:**
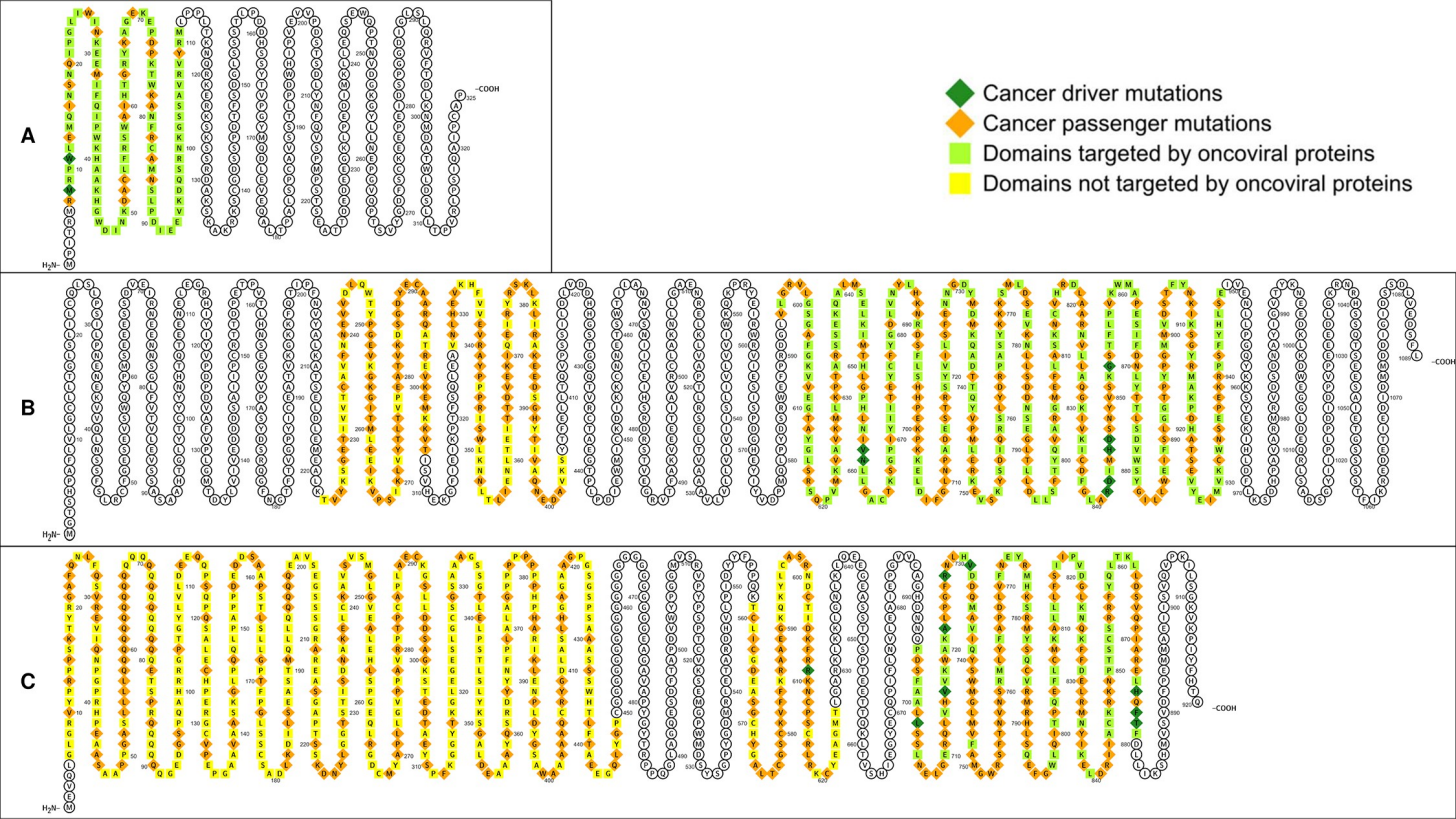
Oncoproteins having at least one oncovirus-targeted domain (OVTD), where driver mutations are either exclusively found or enriched. (A) driver:passenger ratio in oncovirus-targeted PF00605 domain of *IRF1* is higher than the mean driver:passenger ratio for all oncoproteins; (B) driver mutations are exclusively found in oncovirus-targeted PF07714 domain of *PDGFRA*; (C) driver mutations are enriched in oncovirus-targeted PF00104 domain of *AR*.

To confirm that oncoviruses can help identify driver proteins, we first cross-classified human proteins in hvSIN by whether they are oncoviral targets, and whether they are curated by the Cancer Gene Census (CGC) as being causally implicated in cancer, *i*.*e*. driver proteins [76]. Out of 727 oncoviral targets, 93 (12.8%) are in CGC, whereas out of 10897 remaining human proteins in hvSIN, 514 (4.7%) are in CGC. In other words, there is a 3-fold enrichment of driver proteins among oncoviral targets (Fisher’s exact test, two-tailed P = 3 × 10^−16^) (Fig 5A). Next, to find out if oncoviruses can also help identify driver mutations, we cross-classified mutations in oncoproteins by whether they are drivers or passengers, and by whether they map to OVTDs. Oncogenic and resistance mutations with a ClinVar clinical significance value of “pathogenic” or “likely pathogenic” are considered drivers, while passengers include all other missense mutations in oncoproteins that are catalogued by ClinVar and COSMIC. Out of 194 oncoproteins with annotated driver mutations, we identified 30 oncoproteins as having at least one OVTD. Pooled analysis of all 30 oncoproteins mapped 340/398 (85.4%) driver mutations and 3673/7177 (51.2%) passenger mutations to OVTDs. In other words, the odds of finding a driver mutation in OVTDs is 5 times as high as that in non-OVTDs (Fisher’s exact test, two-tailed P < 2.2 × 10^−16^) (Fig 5B). Closer inspection identified 19 candidates for focused investigations into the common basis of viral and mutational oncogenesis (Table 2): (I) 7 oncoproteins where all domains are OVTDs, and the driver:passenger ratio is higher than the average ratio across all oncoproteins; (II) 8 oncoproteins where some domains are OVTDs, and driver mutations are exclusively found in OVTDs; and (III) 4 oncoproteins where some domains are OVTDs, and driver mutations are significantly enriched in OVTDs (Fisher’s exact test, two-tailed P < 0.05). An example of each type of candidate is given in Fig 6.

**Table 2:** Oncoproteins having at least one oncovirus-targeted domain (OVTD), where driver mutations are either exclusively found or enriched.

Type	Oncoprotein	OVTD
I	*BAX*	PF00452
I	*BCL10*	PF00619
I	*CDKN1B*	PF02234
I	*CHEK2*	PF00069; PF00498
I	*IRF1*	PF00605
I	*MAP2K2*	PF00069
I	*PPP2R1A*	PF02985; PF13646
II	*ABL1*	PF00017; PF00018; PF07714
II	*FBXW7*	PF00400
II	*FGFR4*	PF07714
II	*PDGFRA*	PF07714
II	*RAF1*	PF00130; PF07714
II	*RXRA*	PF00104
II	*SPOP*	PF00917
II	*TGFBR2*	PF07714
III	*AR*	PF00104
III	*ATM*	PF00454
III	*RB1*	PF01857
III	*TP53*	PF00870

Type I: All domains are OVTDs, and the driver:passenger ratio is higher than the average ratio across all oncoproteins. Type II: Some domains are OVTDs, and driver mutations are exclusively found in OVTDs. Type III: Some domains are OVTDs, and driver mutations are significantly enriched in OVTDs (Fisher’s exact test, two-tailed P < 0.05).

### Oncovirus-mimicked host domains are enriched for cancer driver mutations

Viruses are known to encode structural homologues that mimic host domains in order to modulate the biological activities of host targets. Such viral homology domains (VHDs) play key roles in mediating immune response (*e*.*g*. PF00048 in CMV and KSHV), apoptosis (*e*.*g*. PF00452 in EBV and KSHV), cell differentiation (*e*.*g*. PF07684 in feline leukemia virus), and protein phosphorylation (*e*.*g*. PF06734 in CMV), among other cellular processes involved in virally-implicated diseases. VHDs often compete with cellular counterparts for interaction partners, thereby rewiring host signaling networks to the virus’s advantage. Table 3 lists instances of human proteins convergently targeted by human domains and oncoviral homology domains in hvSIN.

**Table 3:** Human proteins convergently targeted by human domains and oncoviral homology domains (OVHDs) in hvSIN.

Human domain/OVHD	Pfam description	Human proteins convergently targeted by human domain and OVHD
PF00001	7 transmembrane receptor (rhodopsin family)	*CX3CL1*
PF00017	SH2 domain	*CDC37;* ***HSP90AA1***; ***HSP90AB1****; KHDRBS1;* ***NCKIPSD***; ***PDGFRB***; ***RAF1****; WASL*
PF00018	SH3 domain	*CDC37;* ***HSP90AA1***; ***HSP90AB1****; KHDRBS1;* ***NCKIPSD***; ***PDGFRB****; PPP2CA;* ***RAF1****; WASL*
PF00084	Sushi repeat (SCR repeat)	
PF00134	Cyclin, N-terminal domain	*CCT8; CDK2; CDK3;* ***CDK4****; CDK5;* ***CDK6****; CDK8;* ***CDKN1B***; ***CDKN2A****; POLR2A*
PF00452	Apoptosis regulator proteins, Bcl-2 family	*BAK1;* ***BAX***; ***BCL2****; BCL2L11; BIK; CCDC155; GPX8; PLD3; SPNS1; VRK2*
PF00489	Interleukin-6/G-CSF/MGF family	*IL6R;* ***IL6ST***
PF00605	Interferon regulatory factor transcription factor	
PF00726	Interleukin 10	*IL10RA; IL10RB*
PF01335	Death effector domain	***CASP8****; FADD; RIPK1*
PF07686	Immunoglobulin V-set domain	*NCR3LG1*
PF07714	Protein tyrosine kinase	*CDC37;* ***HSP90AA1***; ***HSP90AB1****; KHDRBS1;* ***NCKIPSD***; ***PDGFRB***; ***RAF1****; WASL*
PF10401	Interferon-regulatory factor 3	***CREBBP***; ***EP300***; ***RB1***

OVHDs are structural homologues of human domains either exclusively occurring in oncoviruses or enriched in oncoviral proteomes (compared to generic viral proteomes). Cancer Gene Census proteins are in bold.

The preceding section established that oncovirus-targeted host domains are enriched for cancer driver mutations. Here, we test the hypothesis that oncovirus-mimicked host domains are also enriched for cancer driver mutations, independent of whether they are physically targeted by the virus. To this end, we identified 21 oncoproteins having at least one oncovirus-targeted domain (OVTD) and at least one viral homology domain (VHD). We further classified viral homology domains (VHDs) into those enriched in oncogenic viruses (oncoviral homology domains, or OVHDs), versus those enriched in non-oncogenic, *i*.*e*. “generic” viruses (generic viral homology domains, or GVHDs) (Methods, S2 Table). We found that domains structurally mimicked by oncoviruses (OVHDs) are more likely to harbour driver mutations, compared to domains structurally mimicked by generic viruses (GVHDs), independent of whether the domain is physically targeted by oncoviruses (OVTD) (CMH test, common odds ratio = 2.2, P = 5 × 10^−5^).

**Fig 7:**
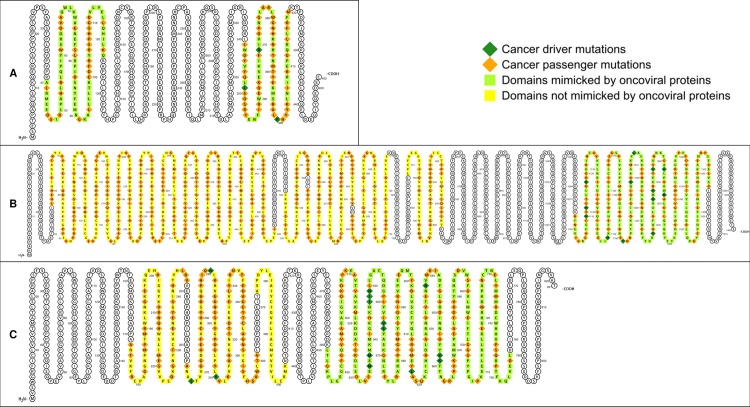
Oncoproteins having no oncovirus-targeted domain (OVTD) but at least one oncoviral homology domain (OVHD), where driver mutations are either exclusively found or enriched. (A) driver:passenger ratio in oncovirus-mimicked PF00178 and PF02198 domains of *ETV6* is higher than the mean driver:passenger ratio for all oncoproteins; (B) driver mutations are exclusively found in oncovirus-mimicked PF07714 domain of *MET*; (C) driver mutations are enriched in oncovirus-mimicked PF07714 domain of *FGFR2*.

We then analyzed the mutational landscape of 44 oncoproteins having at least one oncoviral homology domain (OVHD) but not physically targeted by the virus, *i*.*e*. having no OVTDs. Pooled analysis of all 44 oncoproteins mapped 245/298 (82.2%) driver mutations and 5422/9554 (56.8%) passenger mutations to OVHDs. In other words, the odds of finding a driver mutation in OVHDs is 3 times as high as that in non-OVHDs (Fisher’s exact test, two-tailed P < 2.2 × 10^−16^) (Fig 5B). Closer inspection identified 23 candidates for focused investigations into the common basis of viral and mutational oncogenesis (Table 4): (I) 4 oncoproteins where all domains are OVHDs, and the driver:passenger ratio is higher than the average ratio across all oncoproteins; (II) 16 oncoproteins where some domains are OVHDs, and driver mutations are exclusively found in OVHDs; and (III) 3 oncoproteins where some domains are OVHDs, and driver mutations are significantly enriched in OVHDs (Fisher’s exact test, two-tailed P < 0.05). An example of each type of candidate is given in Fig 7. In summary, oncovirus-mimicked host domains are enriched for cancer driver mutations, regardless of whether these domains are physically targeted by the virus.

**Table 4:** Oncoproteins having no oncovirus-targeted domain (OVTD) but at least one oncoviral homology domain (OVHD), where driver mutations are either exclusively found or enriched.

Type	Oncoprotein	OVHD
I	*ETV6*	PF00178; PF02198
I	*MAX*	PF00010
I	*MC1R*	PF00001
I	*MYC*	PF00010; PF01056; PF02344
II	*AKT3*	PF00169; PF00433
II	*ALK*	PF07714
II	*BTK*	PF00017; PF00018; PF00169; PF07714
II	*ESR1*	PF00104; PF00105
II	*FGFR1*	PF07714
II	*FLT3*	PF07714
II	*KIT*	PF07714
II	*MET*	PF07714
II	*NTRK1*	PF07714
II	*PLCG2*	PF00017; PF00018
II	*POLD1*	PF00136; PF03104
II	*POLE*	PF00136; PF03104
II	*RASA1*	PF00017; PF00018; PF00169
II	*RET*	PF07714
II	*REV3L*	PF00136; PF03104
II	*ROS1*	PF07714
III	*BRAF*	PF07714
III	*ERBB2*	PF07714; PF14843
III	*FGFR2*	PF07714

Type I: All domains are OVHDs, and the driver:passenger ratio is higher than the average ratio across all oncoproteins. Type II: Some domains are OVHDs, and driver mutations are exclusively found in OVHDs. Type III: Some domains are OVHDs, and driver mutations are significantly enriched in OVHDs (Fisher’s exact test, two-tailed P < 0.05).

### Viral proteins and virally-implicated disease mutations tend to perturb the same domain-domain interactions in the human interactome

**Fig 8:**
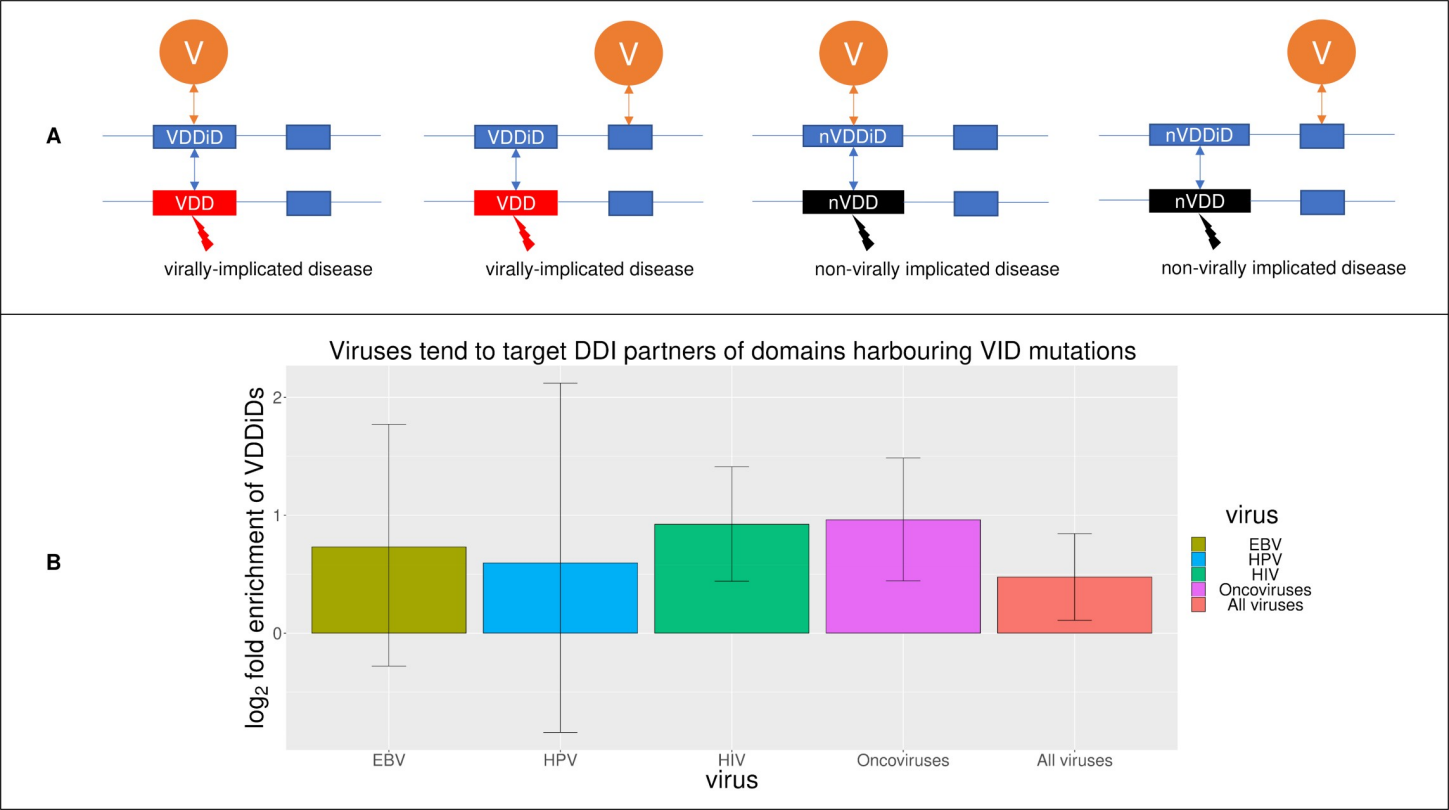
Viral proteins and VID mutations perturb the same domain-domain interactions in the human interactome. (A) From left to right, domains are cross-classified as: interacting with a domain harbouring at least one VID mutation (VDDiD) and targeted by virus, VDDiD not targeted by virus, interacting with a domain harbouring only non-VID mutations (nVDDiD) and targeted by virus, and nVDDiD not targeted by virus. (B) Viruses tend to target VDDiDs rather than nVDDiDs, regardless of whether the VDDiDs and nVDDiDs are susceptible to known disease mutations. The results for EBV and HPV are not statistically significant, possibly due to small sample sizes.

Gulbahce *et al*. previously hypothesized, and established at the whole-protein level, that viruses and VID mutations induce similar perturbations of the human interactome [9]. Here, we test the same hypothesis at the higher resolution of protein domains, by examining whether viruses and VID mutations perturb the same domain-domain interactions (DDIs) in the human interactome. In other words, do viruses tend to target DDI partners of domains harbouring VID mutations (viral disease domain-interacting domains, or VDDiDs), rather than DDI partners of domains harbouring non-VID mutations (non-viral disease domain-interacting domains, or nVDDiDs) (Fig 8A)? As some domains can interact with both VID domains and non-VID domains, we define VDDiDs as domains that interact with at least one VID domain, and nVDDiDs as domains that exclusively interact with non-VID domains. We found that EBV and HPV exhibit a slight preference for targeting VDDiDs, although the effect sizes are not statistically significant (42/62 VDDiDs *vs*. 58/104 nVDDiDs for EBV, and 20/29 VDDiDs *vs*. 41/69 nVDDiDs for HPV). HIV targets 218/309 (70.6%) VDDiDs and 193/346 (55.8%) nVDDiDs, representing a 1.9-fold enrichment of VDDiDs among HIV-targeted domains (Fisher’s exact test, two-tailed P = 1 × 10^−4^). Similarly, oncoviruses target 204/285 (71.6%) VDDiDs and 164/291 (56.4%) nVDDiDs, *i*.*e*. a 1.9-fold enrichment of VDDiDs among oncovirus-targeted domains (Fisher’s exact test, two-tailed P = 1 × 10^−4^). Finally, a meta-analysis on the common effect of all viral proteins and all mutations causing proliferative and immunological diseases found that viruses target 424/599 (70.8%) VDDiDs and 350/551 (63.5%) nVDDiDs, *i*.*e*. a 1.4-fold enrichment of VDDiDs among virus-targeted domains (Fisher’s exact test, two-tailed P = 0.01) (Fig 8B).

**Fig 9:**
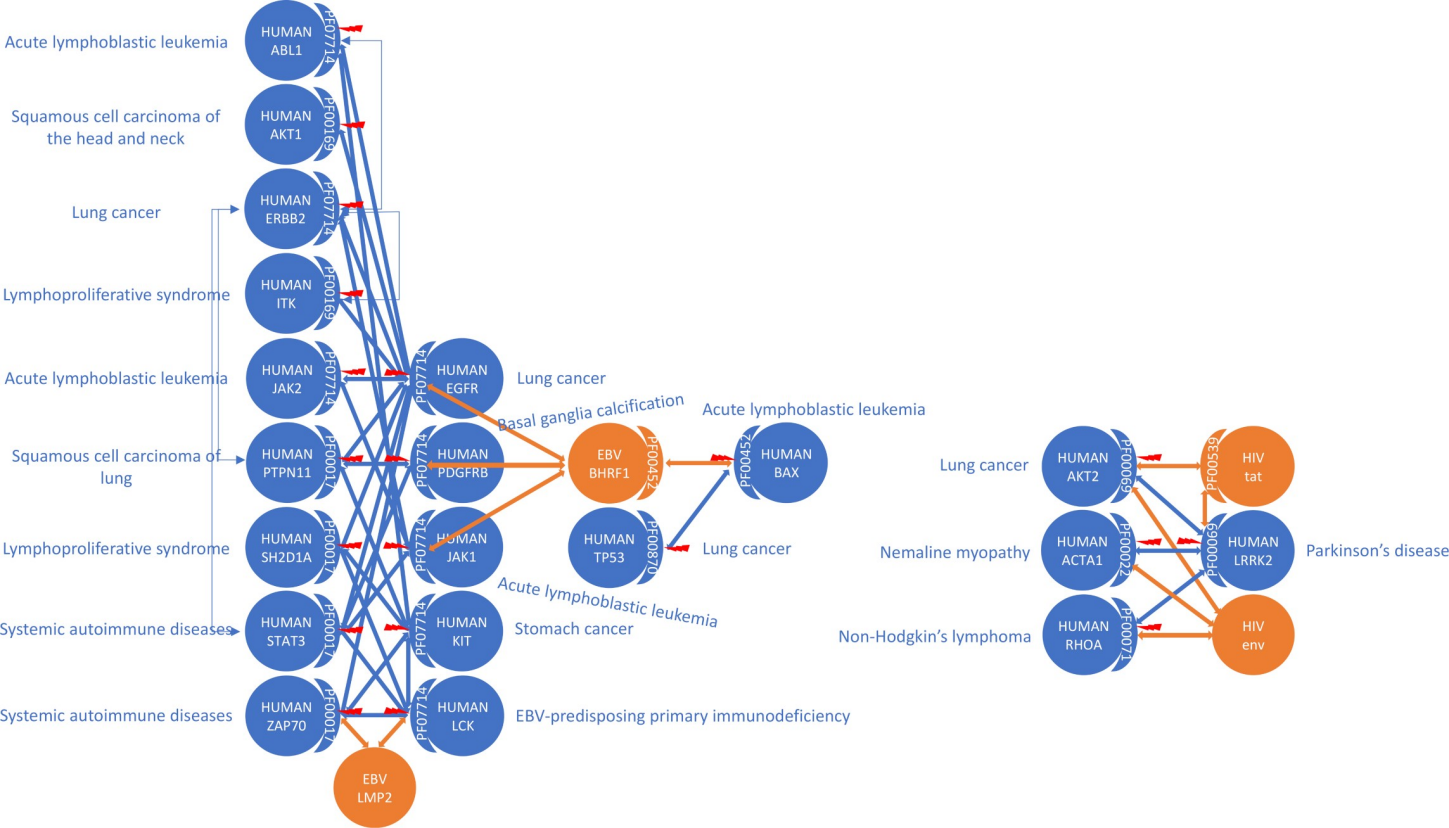
Viral proteins and VID mutations convergently perturb dense regions of the human domain interactome. Examples are given for EBV (left) and HIV (right).

Virus’s preferential targeting of VDDiDs may be confounded by the tendency for viruses to target VID domains (Fig 2), and the tendency for VID domains to interact among themselves. We therefore excluded domains susceptible to known disease mutations and examined the extent to which virus targets “non-disease” domains that interact with VID domains. We found that HIV targets 179/250 (71.6%) VDDiDs and 164/285 (57.5%) nVDDiDs that do not harbour any known disease mutation (Fisher’s exact test odds ratio = 1.9, two-tailed P = 8 × 10^−4^). Similarly, oncoviruses target 165/230 (71.7%) VDDiDs and 137/237 (57.8%) nVDDiDs that do not harbour any known disease mutation (Fisher’s exact test odds ratio = 1.8, two-tailed P = 2 × 10^−3^). Pooled analysis of all viruses found that overall, viruses target 345/481 (71.7%) VDDiDs and 295/456 (64.7%) nVDDiDs that do not harbour any known disease mutation (Fisher’s exact test odds ratio = 1.4, two-tailed P = 0.02). Virus’s preferential targeting of VDDiDs supports our hypothesis that viruses and VID mutations inducing similar disease phenotypes convergently perturb the host domain interactome, possibly unveiling core disease modules underlying clinically heterogeneous virally-implicated diseases (Fig 9).

## Discussion

Structural interaction networks serve as a valuable tool for understanding the molecular mechanisms of genetic diseases, as well as the fundamental differences between endogenous and exogenous PPI networks. As experimental determination of protein structure remains an arduous task, homology modelling offers an efficient alternative for the structural annotation of protein complexes. This is based on the observation that PPIs are often mediated by evolutionarily conserved structural modules, such as domains and short linear motifs [77]. Here, we reassess the role of viral proteins as surrogates for human disease variants in relating interactome network perturbation to disease phenotypes, using a domain-resolved human-virus protein interactome where human domains are annotated with disease variant information. Compared to previous work demonstrating general proximity between viral targets and VID proteins in the human interactome, our results provide a structural explanation for the equivalent pathogenicity of viral proteins and VID mutations. Whereas previous studies merely recognized the existence of viral homologues of cellular domains, we delve deeper into the functional implications of oncoviral domain homology. Our approach can readily identify domains convergently targeted or mimicked by diverse oncoviruses for focused screening of driver mutations across various types of cancer. Further characterization of cellular domains and motifs interacting with domains targeted or mimicked by viruses may uncover immune evasion strategies exploited in common by cancer cells and pathogens, and shed light on pathways dysregulated in other virally-implicated disorders.

Although most of our findings are statistically significant, there are notable differences in the enrichment of VID mutations in virus-targeted domains, both among individual viruses (EBV, HPV and HIV), as well as between single-virus analysis and pooled analysis on multiple viruses. For single-virus analysis, enrichment effect size and significance are impacted by the number of virus-host protein-protein interactions and virus-specific diseases, which ultimately determine the statistical power. Pooled analysis on all oncoviruses detected trends in the same direction as analysis on single oncoviruses (EBV and HPV), but with higher statistical power. In addition to investigator bias resulting in some viruses having a higher number of mapped virus-host PPIs, it is also possible that certain viruses prefer to perturb host regulatory network, rather than host PPI network, which is beyond the scope of this work. Compared to direct targeting of VID domains (a “first-degree” effect), viral targeting of the interaction partners of VID domains is expected to have a weaker, “second-degree” effect on the VID domains. This partly explains why results of the “first-degree” analysis on EBV and HPV (Fig 2) are stronger than those of the “second-degree” analysis (Fig 8B).

Our pooled analysis of all oncoviral targets and all oncomutations is motivated by the assumption of convergent evolution and mimicry of endogenous oncogenic mechanisms by diverse oncoviruses. There is compelling evidence of different oncoviruses complementing each other’s replication and persistence strategies, thus eliciting multiple cellular responses associated with the hallmarks of cancer. One example is primary effusion lymphoma, a disease causally linked to KSHV but also having an EBV component. While expression of KSHV lytic genes such as *vIL-6* and *K1* promote VEGF secretion and angiogenesis, concomitant expression of EBV latent genes confers additional anti-apoptotic properties to infected cells in the initial phase of lymphomagenesis [78, 79]. Given the paucity of context-dependent (*i*.*e*. tissue- and disease-specific) host-endogenous and host-pathogen PPI data, here we focus on establishing viral proteins and genetic mutations that induce similar disease phenotypes as generally equivalent perturbagens of the human interactome. Future work will also consider the diversity of host range and tissue tropism among different viruses, and the potentially distinct functional impacts of the same mutation in different cell types and diseases.

One potential caveat of our interactome perturbation model is its incompleteness, due to the following reasons. Firstly, current mapping of the host-virus protein interactome is far from exhaustive. Secondly, some bona fide host-virus PPIs cannot be modelled by existing domain-based interaction templates. Thirdly, virus may not interact with a host protein via PPI, but rather regulate its expression via transcriptional or epigenetic mechanisms. Lastly, our study only considers missense mutations, because domain-based analysis of interactome perturbation requires precise positioning of mutations with respect to protein domains. Missense mutations can be unambiguously mapped to individual domains, whereas other types of mutations (*e*.*g*. nonsense or frameshift) may cause more drastic changes in the protein structure and are more difficult to map to individual domains. We are aware, however, of literature suggesting that nonsense and frameshift mutations tend to occur more frequently in tumour suppressor genes than in oncogenes [80]. Effects of these mutations on the integrity of the human interactome warrant further investigation. Still, despite the incompleteness of our model, we observed significant convergent perturbation of the human domain-resolved interactome by viruses and mutations inducing similar disease phenotypes.

The advent of high-throughput biotechnology has made it possible to comprehensively characterize genomic variations in and interspecies interactions between human and microbes, which play important roles in health and disease. As more data on pathogen-implicated diseases and host-pathogen interactions emerge, our approach may be extended to the study of bacterial diseases and co-infections involving multiple pathogenic species, such as the co-pathogenesis of HIV and Mycobacterium tuberculosis. By combining these data within the framework of structural systems biology, our work sets the stage for multi-scale, integrative investigations into endogenous and exogenous perturbagens of the human interactome, thus helping to elucidate the molecular mechanisms of infection and its possible connections to genetic diseases such as cancer, autoimmunity, and neurodegeneration.

## Methods

### Construction of disease-annotated human-virus structural interaction network

Human-endogenous and human-virus binary PPI data were obtained from IntAct [14], HPIDB [15], and the HIV-1 Human Interaction Database [16–18]. Structural templates for domain-domain and domain-motif interactions were obtained from 3did [19], iPfam [21] and ELM [20]. Protein sequences were scanned for Pfam domains using InterProScan under default settings (version 5.30–69.0) [23, 81], and for the occurrence of domain-binding motifs as defined by 3did and ELM. Domain-based interaction models were assigned to each PPI by extracting all DDIs and DMIs possibly mediating the PPI. Disease association and clinical significance of variants were obtained from UniProtKB, ClinVar, and COSMIC [24, 25, 76]. Ensembl Variant Effect Predictor (VEP v93.0) was used for extracting variant genomic location, variation class, reference allele, HGVS notations, amino acid position, overlapping Pfam domains, among other features [82]. To facilitate counting of mutational events, variants are annotated with RefSNP IDs using VEP’s check_existing flag. Variants not co-located with any known variant are merged based on identical genomic location, variation class, and shared alleles, as per NCBI guidelines for merging submitted SNPs into RefSNP clusters (https://www.ncbi.nlm.nih.gov/books/NBK44417/). Only missense mutations located inside Pfam domains were retained for analyses. Assignment of each virally-implicated disease (VID) to EBV, HPV and HIV was based on at least two literature sources (S1 Text). To minimize redundancy in disease annotation, UMLS and OMIM IDs given to subtypes of the same disease were merged into the more general Disease Ontology [83], Orphanet [84] and MeSH IDs.

### Pooled analysis of viral proteins and disease mutations

Oncoviruses are as classified by CDC, IARC, and MeSH (https://www.ncbi.nlm.nih.gov/mesh/68009858). Cancer is defined as any disease whose parent terms include “DOID:162”, “ORPHA:250908”, or MeSH IDs beginning with “C04.557|C04.588|C04.619|C04.626|C04.651|C04.666|C04.682|C04.692|C04.697|C04.700|C04.730|C04.834|C04.850”. Diseases without Disease Ontology, Orphanet or MeSH IDs are manually labelled as “cancer” if their names match the following regular expression: “blastoma|cancer|carcino*|glioma|leukemia|leukaemia|lymphoma|melanoma|neoplas*|sarcoma|tumour|tumor”. Proliferative diseases have parent terms “DOID:14566”, “ORPHA:250908”, or MeSH IDs beginning with “C04”. Immunological diseases have parent terms “DOID:2914”, “ORPHA:98004”, or MeSH IDs beginning with “C20”. All statistical analyses were conducted in R [85]. Plots of domain-level distribution of disease mutations were created with Protter [86].

### Classification of viral homology domains

Pfam domain annotation for all human and viral proteins in UniProt was retrieved from InterPro (Release 69.0) [87]. We define viral homology domains (VHDs) as Pfam domains conserved between human and viral proteins. For each VHD, the likelihood of it occurring in oncoviruses was calculated as the number of oncoviruses encoding the VHD, divided by the total number of unique oncoviral species in UniProt. Similarly, the likelihood of a VHD occurring in “generic” (*i*.*e*. non-oncogenic) viruses was calculated as the number of generic viruses encoding the VHD divided by the total number of unique generic viral species in UniProt. The observed likelihood ratio (LR) of an oncovirus *vs*. a generic virus encoding the VHD is then the ratio of the two likelihoods. We then permuted the label “oncovirus” and “generic virus” 10000 times among viruses encoding the VHD, thereby obtaining a null distribution for the LR. An empirical p-value for the enrichment or depletion of a VHD in oncoviral proteomes was calculated according to [88]. VHDs whose observed LR > 1 and Benjamini-Hochberg adjusted p-values (q-values) < 0.1 are considered enriched in oncoviral proteomes. These VHDs and other VHDs exclusively occurring in oncoviruses are called oncoviral homology domains (OVHDs). Likewise, VHDs whose observed LR < 1 and q-values < 0.1 are considered enriched in generic viral proteomes. These VHDs and other VHDs exclusively occurring in generic viruses are called generic viral homology domains (GVHDs).

## Supporting information

S1 Text References for virally implicated diseases. (DOCX)

S1 Table Virally implicated diseases and disease-associated proteins for EBV, HPV and HIV. Disease proteins are shown in brackets if they are targeted by virus, but the human-virus PPI does not have a domain-based structural model. Disease proteins are in bold if the domain harbouring disease mutation is targeted by virus.
(XLSX)

S2 Table Human domains exclusively occurring or enriched in oncoviruses vs. generic viruses. For each Pfam domain, a human instance (UniProt ID) and a viral instance (Taxonomy ID|Taxonomy Name|UniProt ID) are selected, with preference given to proteins in hvSIN. Mappings of Pfam domains to Gene Ontology terms are obtained from http://geneontology.org/external2go/pfam2go.
(CSV)
