# Supplementary material for: Convergent perturbation of the human domain-resolved interactome by viruses and mutations inducing similar disease phenotypes
Source: PLoS Comput Biol. 2019 Feb 13;15(2):e1006762. doi: 10.1371/journal.pcbi.1006762 (PMC6373925; doi:10.1371/journal.pcbi.1006762)
Supplement: S1 Text — (DOCX) [file pcbi.1006762.s001.docx]

**References for virally implicated diseases**

EBV: Acute lymphoblastic leukemia [1-4], Basal ganglia calcification [5-7], EBV disease-predisposing primary immunodeficiencies [8-13], Hemophagocytic lymphohistiocytosis [14-19], Histiocytic medullary reticulosis [20-23], Inflammatory bowel disease [24-29], Lung cancer [30-34], Lymphoma [35-44], Lymphoproliferative syndromes [45-49], Nasopharyngeal carcinoma [50-55], Stomach cancer [56-61], Systemic autoimmune diseases [62-67]

HPV: Bladder cancer [68-70], Breast cancer [71-73], Cervical cancer [74-76], Colorectal cancer [77-79], Lung cancer [80-82], Ovarian cancer [83-85], Prostate cancer [86-88], Squamous cell carcinoma of head and neck [89-91], Vulvar cancer [92-94]

HIV: Acute myeloid leukemia [95-97], Cervical/Liver/Lung/Testicular cancer and Lymphoma [98-103], Dilated cardiomyopathy [104-106], Long QT syndrome [107-109], Myelodysplastic syndrome [110, 111], Nemaline myopathy [112-114], Parkinson’s disease [115-117], Pulmonary arterial hypertension [118-121]

1. Ateyah ME, Hashem ME, Abdelsalam M. Epstein-Barr virus and regulatory T cells in Egyptian paediatric patients with acute B lymphoblastic leukaemia. J Clin Pathol. 2017;70(2):120-5.

2. Sehgal S, Mujtaba S, Gupta D, Aggarwal R, Marwaha RK. High incidence of Epstein Barr virus infection in childhood acute lymphocytic leukemia: a preliminary study. Indian J Pathol Microbiol. 2010;53(1):63-7.

3. Hermouet S, Sutton CA, Rose TM, Greenblatt RJ, Corre I, Garand R, et al. Qualitative and quantitative analysis of human herpesviruses in chronic and acute B cell lymphocytic leukemia and in multiple myeloma. Leukemia. 2003;17(1):185-95.

4. Sakajiri S, Mori K, Isobe Y, Kawamata N, Oshimi K. Epstein-Barr virus-associated T-cell acute lymphoblastic leukaemia. Br J Haematol. 2002;117(1):127-9.

5. Ishikawa N, Kawaguchi H, Nakamura K, Kobayashi M. Central nervous system complications and neuroradiological findings in children with chronic active Epstein-Barr virus infection. Pediatr Int. 2013;55(1):72-8.

6. Kimura H, Hoshino Y, Kanegane H, Tsuge I, Okamura T, Kawa K, et al. Clinical and virologic characteristics of chronic active Epstein-Barr virus infection. Blood. 2001;98(2):280-6.

7. Morita M, Tsuge I, Matsuoka H, Ito Y, Itosu T, Yamamoto M, et al. Calcification in the basal ganglia with chronic active Epstein-Barr virus infection. Neurology. 1998;50(5):1485-8.

8. Picard C, Al-Herz W, Bousfiha A, Casanova JL, Chatila T, Conley ME, et al. Primary Immunodeficiency Diseases: an Update on the Classification from the International Union of Immunological Societies Expert Committee for Primary Immunodeficiency 2015. J Clin Immunol. 2015;35(8):696-726.

9. Houldcroft CJ, Kellam P. Host genetics of Epstein-Barr virus infection, latency and disease. Rev Med Virol. 2015;25(2):71-84.

10. Li FY, Chaigne-Delalande B, Su H, Uzel G, Matthews H, Lenardo MJ. XMEN disease: a new primary immunodeficiency affecting Mg2+ regulation of immunity against Epstein-Barr virus. Blood. 2014;123(14):2148-52.

11. Parvaneh N, Filipovich AH, Borkhardt A. Primary immunodeficiencies predisposed to Epstein-Barr virus-driven haematological diseases. Br J Haematol. 2013;162(5):573-86.

12. Katano H, Ali MA, Patera AC, Catalfamo M, Jaffe ES, Kimura H, et al. Chronic active Epstein-Barr virus infection associated with mutations in perforin that impair its maturation. Blood. 2004;103(4):1244-52.

13. Coffey AJ, Brooksbank RA, Brandau O, Oohashi T, Howell GR, Bye JM, et al. Host response to EBV infection in X-linked lymphoproliferative disease results from mutations in an SH2-domain encoding gene. Nat Genet. 1998;20(2):129-35.

14. Kawamura Y, Miura H, Matsumoto Y, Uchida H, Kudo K, Hata T, et al. A case of Epstein-Barr virus-associated hemophagocytic lymphohistiocytosis with severe cardiac complications. BMC Pediatr. 2016;16(1):172.

15. Smith MC, Cohen DN, Greig B, Yenamandra A, Vnencak-Jones C, Thompson MA, et al. The ambiguous boundary between EBV-related hemophagocytic lymphohistiocytosis and systemic EBV-driven T cell lymphoproliferative disorder. Int J Clin Exp Pathol. 2014;7(9):5738-49.

16. Janka GE. Familial and acquired hemophagocytic lymphohistiocytosis. Annu Rev Med. 2012;63:233-46.

17. Rohr J, Beutel K, Maul-Pavicic A, Vraetz T, Thiel J, Warnatz K, et al. Atypical familial hemophagocytic lymphohistiocytosis due to mutations in UNC13D and STXBP2 overlaps with primary immunodeficiency diseases. Haematologica. 2010;95(12):2080-7.

18. Henter JI, Ehrnst A, Andersson J, Elinder G. Familial hemophagocytic lymphohistiocytosis and viral infections. Acta Paediatr. 1993;82(4):369-72.

19. Su IJ, Hsu YH, Lin MT, Cheng AL, Wang CH, Weiss LM. Epstein-Barr virus-containing T-cell lymphoma presents with hemophagocytic syndrome mimicking malignant histiocytosis. Cancer. 1993;72(6):2019-27.

20. Chen RL, Su IJ, Lin KH, Lee SH, Lin DT, Chuu WM, et al. Fulminant childhood hemophagocytic syndrome mimicking histiocytic medullary reticulosis. An atypical form of Epstein-Barr virus infection. Am J Clin Pathol. 1991;96(2):171-6.

21. Su IJ, Hsieh HJ, Lee CY. Histiocytic medullary reticulosis: a lethal form of primary EBV infection in young children in Taiwan. Lancet. 1989;1(8634):389.

22. Rosner F, Grunwald HW. Association of T cell acute lymphoblastic leukemia and histiocytic medullary reticulosis. Am J Med. 1984;77(5):910-4.

23. Cohen RA, Hutter JJ, Jr., Boxer MA, Goldman DS. Histiocytic medullary reticulosis associated with acute Epstein-Barr (EB) virus infection. Am J Pediatr Hematol Oncol. 1980;2(3):245-8.

24. Wang W, Jovel J, Halloran B, Wine E, Patterson J, Ford G, et al. Metagenomic analysis of microbiome in colon tissue from subjects with inflammatory bowel diseases reveals interplay of viruses and bacteria. Inflamm Bowel Dis. 2015;21(6):1419-27.

25. Nissen LH, Nagtegaal ID, de Jong DJ, Kievit W, Derikx LA, Groenen PJ, et al. Epstein-Barr virus in inflammatory bowel disease: the spectrum of intestinal lymphoproliferative disorders. J Crohns Colitis. 2015;9(5):398-403.

26. Magro F, Santos-Antunes J, Albuquerque A, Vilas-Boas F, Macedo GN, Nazareth N, et al. Epstein-Barr virus in inflammatory bowel disease-correlation with different therapeutic regimens. Inflamm Bowel Dis. 2013;19(8):1710-6.

27. Dimitroulia E, Pitiriga VC, Piperaki ET, Spanakis NE, Tsakris A. Inflammatory bowel disease exacerbation associated with Epstein-Barr virus infection. Dis Colon Rectum. 2013;56(3):322-7.

28. Spieker T, Herbst H. Distribution and phenotype of Epstein-Barr virus-infected cells in inflammatory bowel disease. Am J Pathol. 2000;157(1):51-7.

29. Yanai H, Shimizu N, Nagasaki S, Mitani N, Okita K. Epstein-Barr virus infection of the colon with inflammatory bowel disease. Am J Gastroenterol. 1999;94(6):1582-6.

30. Wang S, Xiong H, Yan S, Wu N, Lu Z. Identification and Characterization of Epstein-Barr Virus Genomes in Lung Carcinoma Biopsy Samples by Next-Generation Sequencing Technology. Sci Rep. 2016;6:26156.

31. Gomez-Roman JJ, Martinez MN, Fernandez SL, Val-Bernal JF. Epstein-Barr virus-associated adenocarcinomas and squamous-cell lung carcinomas. Mod Pathol. 2009;22(4):530-7.

32. Chen FF, Yan JJ, Lai WW, Jin YT, Su IJ. Epstein-Barr virus-associated nonsmall cell lung carcinoma: undifferentiated "lymphoepithelioma-like" carcinoma as a distinct entity with better prognosis. Cancer. 1998;82(12):2334-42.

33. Wong MP, Chung LP, Yuen ST, Leung SY, Chan SY, Wang E, et al. In situ detection of Epstein-Barr virus in non-small cell lung carcinomas. J Pathol. 1995;177(3):233-40.

34. Kasai K, Sato Y, Kameya T, Inoue H, Yoshimura H, Kon S, et al. Incidence of latent infection of Epstein-Barr virus in lung cancers--an analysis of EBER1 expression in lung cancers by in situ hybridization. J Pathol. 1994;174(4):257-65.

35. Navari M, Fuligni F, Laginestra MA, Etebari M, Ambrosio MR, Sapienza MR, et al. Molecular signature of Epstein Barr virus-positive Burkitt lymphoma and post-transplant lymphoproliferative disorder suggest different roles for Epstein Barr virus. Front Microbiol. 2014;5:728.

36. Klein E, Kis LL, Klein G. Epstein-Barr virus infection in humans: from harmless to life endangering virus-lymphocyte interactions. Oncogene. 2007;26(9):1297-305.

37. Klumb CE, Hassan R, De Oliveira DE, De Resende LM, Carrico MK, De Almeida Dobbin J, et al. Geographic variation in Epstein-Barr virus-associated Burkitt's lymphoma in children from Brazil. Int J Cancer. 2004;108(1):66-70.

38. Tao Q, Robertson KD, Manns A, Hildesheim A, Ambinder RF. Epstein-Barr virus (EBV) in endemic Burkitt's lymphoma: molecular analysis of primary tumor tissue. Blood. 1998;91(4):1373-81.

39. Epstein MA, Achong BG, Barr YM. Virus Particles in Cultured Lymphoblasts from Burkitt's Lymphoma. Lancet. 1964;1(7335):702-3.

40. Rowe M, Raithatha S, Shannon-Lowe C. Counteracting effects of cellular Notch and Epstein-Barr virus EBNA2: implications for stromal effects on virus-host interactions. J Virol. 2014;88(20):12065-76.

41. Heslop HE. Biology and treatment of Epstein-Barr virus-associated non-Hodgkin lymphomas. Hematology Am Soc Hematol Educ Program. 2005:260-6.

42. van Baarle D, Hovenkamp E, Callan MF, Wolthers KC, Kostense S, Tan LC, et al. Dysfunctional Epstein-Barr virus (EBV)-specific CD8(+) T lymphocytes and increased EBV load in HIV-1 infected individuals progressing to AIDS-related non-Hodgkin lymphoma. Blood. 2001;98(1):146-55.

43. Boyle MJ, Sewell WA, Sculley TB, Apolloni A, Turner JJ, Swanson CE, et al. Subtypes of Epstein-Barr virus in human immunodeficiency virus-associated non-Hodgkin lymphoma. Blood. 1991;78(11):3004-11.

44. Jones JF, Shurin S, Abramowsky C, Tubbs RR, Sciotto CG, Wahl R, et al. T-cell lymphomas containing Epstein-Barr viral DNA in patients with chronic Epstein-Barr virus infections. N Engl J Med. 1988;318(12):733-41.

45. Cai Q, Chen K, Young KH. Epstein-Barr virus-positive T/NK-cell lymphoproliferative disorders. Exp Mol Med. 2015;47:e133.

46. Ok CY, Li L, Young KH. EBV-driven B-cell lymphoproliferative disorders: from biology, classification and differential diagnosis to clinical management. Exp Mol Med. 2015;47:e132.

47. Kimura H, Ito Y, Kawabe S, Gotoh K, Takahashi Y, Kojima S, et al. EBV-associated T/NK-cell lymphoproliferative diseases in nonimmunocompromised hosts: prospective analysis of 108 cases. Blood. 2012;119(3):673-86.

48. Carbone A, Gloghini A, Dotti G. EBV-associated lymphoproliferative disorders: classification and treatment. Oncologist. 2008;13(5):577-85.

49. Quintanilla-Martinez L, Kumar S, Fend F, Reyes E, Teruya-Feldstein J, Kingma DW, et al. Fulminant EBV(+) T-cell lymphoproliferative disorder following acute/chronic EBV infection: a distinct clinicopathologic syndrome. Blood. 2000;96(2):443-51.

50. Lung ML, Cheung AK, Ko JM, Lung HL, Cheng Y, Dai W. The interplay of host genetic factors and Epstein-Barr virus in the development of nasopharyngeal carcinoma. Chin J Cancer. 2014;33(11):556-68.

51. Cao JY, Mansouri S, Frappier L. Changes in the nasopharyngeal carcinoma nuclear proteome induced by the EBNA1 protein of Epstein-Barr virus reveal potential roles for EBNA1 in metastasis and oxidative stress responses. J Virol. 2012;86(1):382-94.

52. Lo YM, Chan LY, Chan AT, Leung SF, Lo KW, Zhang J, et al. Quantitative and temporal correlation between circulating cell-free Epstein-Barr virus DNA and tumor recurrence in nasopharyngeal carcinoma. Cancer Res. 1999;59(21):5452-5.

53. Nicholls JM, Agathanggelou A, Fung K, Zeng X, Niedobitek G. The association of squamous cell carcinomas of the nasopharynx with Epstein-Barr virus shows geographical variation reminiscent of Burkitt's lymphoma. J Pathol. 1997;183(2):164-8.

54. Young LS, Dawson CW, Clark D, Rupani H, Busson P, Tursz T, et al. Epstein-Barr virus gene expression in nasopharyngeal carcinoma. J Gen Virol. 1988;69 ( Pt 5):1051-65.

55. de Schryver A, Friberg S, Jr., Klein G, Henle W, Henle G, De-The G, et al. Epstein-Barr virus-associated antibody patterns in carcinoma of the post-nasal space. Clin Exp Immunol. 1969;5(5):443-59.

56. Abe H, Kaneda A, Fukayama M. Epstein-Barr Virus-Associated Gastric Carcinoma: Use of Host Cell Machineries and Somatic Gene Mutations. Pathobiology. 2015;82(5):212-23.

57. Niller HH, Szenthe K, Minarovits J. Epstein-Barr virus-host cell interactions: an epigenetic dialog? Front Genet. 2014;5:367.

58. Iizasa H, Nanbo A, Nishikawa J, Jinushi M, Yoshiyama H. Epstein-Barr Virus (EBV)-associated gastric carcinoma. Viruses. 2012;4(12):3420-39.

59. Camargo MC, Murphy G, Koriyama C, Pfeiffer RM, Kim WH, Herrera-Goepfert R, et al. Determinants of Epstein-Barr virus-positive gastric cancer: an international pooled analysis. Br J Cancer. 2011;105(1):38-43.

60. Fukayama M, Hino R, Uozaki H. Epstein-Barr virus and gastric carcinoma: virus-host interactions leading to carcinoma. Cancer Sci. 2008;99(9):1726-33.

61. Shibata D, Weiss LM. Epstein-Barr virus-associated gastric adenocarcinoma. Am J Pathol. 1992;140(4):769-74.

62. Draborg AH, Duus K, Houen G. Epstein-Barr virus in systemic autoimmune diseases. Clin Dev Immunol. 2013;2013:535738.

63. Munz C, Lunemann JD, Getts MT, Miller SD. Antiviral immune responses: triggers of or triggered by autoimmunity? Nat Rev Immunol. 2009;9(4):246-58.

64. Serafini B, Rosicarelli B, Franciotta D, Magliozzi R, Reynolds R, Cinque P, et al. Dysregulated Epstein-Barr virus infection in the multiple sclerosis brain. J Exp Med. 2007;204(12):2899-912.

65. Lunemann JD, Kamradt T, Martin R, Munz C. Epstein-barr virus: environmental trigger of multiple sclerosis? J Virol. 2007;81(13):6777-84.

66. Poole BD, Scofield RH, Harley JB, James JA. Epstein-Barr virus and molecular mimicry in systemic lupus erythematosus. Autoimmunity. 2006;39(1):63-70.

67. Harley JB, Chen X, Pujato M, Miller D, Maddox A, Forney C, et al. Transcription factors operate across disease loci, with EBNA2 implicated in autoimmunity. Nat Genet. 2018;50(5):699-707.

68. Li N, Yang L, Zhang Y, Zhao P, Zheng T, Dai M. Human papillomavirus infection and bladder cancer risk: a meta-analysis. J Infect Dis. 2011;204(2):217-23.

69. Furihata M, Inoue K, Ohtsuki Y, Hashimoto H, Terao N, Fujita Y. High-risk human papillomavirus infections and overexpression of p53 protein as prognostic indicators in transitional cell carcinoma of the urinary bladder. Cancer Res. 1993;53(20):4823-7.

70. Anwar K, Naiki H, Nakakuki K, Inuzuka M. High frequency of human papillomavirus infection in carcinoma of the urinary bladder. Cancer. 1992;70(7):1967-73.

71. Salman NA, Davies G, Majidy F, Shakir F, Akinrinade H, Perumal D, et al. Association of High Risk Human Papillomavirus and Breast cancer: A UK based Study. Sci Rep. 2017;7:43591.

72. Li N, Bi X, Zhang Y, Zhao P, Zheng T, Dai M. Human papillomavirus infection and sporadic breast carcinoma risk: a meta-analysis. Breast Cancer Res Treat. 2011;126(2):515-20.

73. Kan CY, Iacopetta BJ, Lawson JS, Whitaker NJ. Identification of human papillomavirus DNA gene sequences in human breast cancer. Br J Cancer. 2005;93(8):946-8.

74. Smith JS, Lindsay L, Hoots B, Keys J, Franceschi S, Winer R, et al. Human papillomavirus type distribution in invasive cervical cancer and high-grade cervical lesions: a meta-analysis update. Int J Cancer. 2007;121(3):621-32.

75. Schiffman M, Castle PE, Jeronimo J, Rodriguez AC, Wacholder S. Human papillomavirus and cervical cancer. Lancet. 2007;370(9590):890-907.

76. Walboomers JM, Jacobs MV, Manos MM, Bosch FX, Kummer JA, Shah KV, et al. Human papillomavirus is a necessary cause of invasive cervical cancer worldwide. J Pathol. 1999;189(1):12-9.

77. Baandrup L, Thomsen LT, Olesen TB, Andersen KK, Norrild B, Kjaer SK. The prevalence of human papillomavirus in colorectal adenomas and adenocarcinomas: a systematic review and meta-analysis. Eur J Cancer. 2014;50(8):1446-61.

78. Damin DC, Ziegelmann PK, Damin AP. Human papillomavirus infection and colorectal cancer risk: a meta-analysis. Colorectal Dis. 2013;15(8):e420-8.

79. Bodaghi S, Yamanegi K, Xiao SY, Da Costa M, Palefsky JM, Zheng ZM. Colorectal papillomavirus infection in patients with colorectal cancer. Clin Cancer Res. 2005;11(8):2862-7.

80. Klein F, Amin Kotb WF, Petersen I. Incidence of human papilloma virus in lung cancer. Lung Cancer. 2009;65(1):13-8.

81. Syrjanen KJ. HPV infections and lung cancer. J Clin Pathol. 2002;55(12):885-91.

82. Cheng YW, Chiou HL, Sheu GT, Hsieh LL, Chen JT, Chen CY, et al. The association of human papillomavirus 16/18 infection with lung cancer among nonsmoking Taiwanese women. Cancer Res. 2001;61(7):2799-803.

83. Roos P, Orlando PA, Fagerstrom RM, Pepper JW. In North America, some ovarian cancers express the oncogenes of preventable human papillomavirus HPV-18. Sci Rep. 2015;5:8645.

84. Rosa MI, Silva GD, de Azedo Simoes PW, Souza MV, Panatto AP, Simon CS, et al. The prevalence of human papillomavirus in ovarian cancer: a systematic review. Int J Gynecol Cancer. 2013;23(3):437-41.

85. Wu QJ, Guo M, Lu ZM, Li T, Qiao HZ, Ke Y. Detection of human papillomavirus-16 in ovarian malignancy. Br J Cancer. 2003;89(4):672-5.

86. Yin B, Liu W, Yu P, Liu C, Chen Y, Duan X, et al. Association between human papillomavirus and prostate cancer: A meta-analysis. Oncol Lett. 2017;14(2):1855-65.

87. Singh N, Hussain S, Kakkar N, Singh SK, Sobti RC, Bharadwaj M. Implication of high risk human papillomavirus HR-HPV infection in prostate cancer in Indian population--a pioneering case-control analysis. Sci Rep. 2015;5:7822.

88. Pascale M, Pracella D, Barbazza R, Marongiu B, Roggero E, Bonin S, et al. Is human papillomavirus associated with prostate cancer survival? Dis Markers. 2013;35(6):607-13.

89. Nulton TJ, Olex AL, Dozmorov M, Morgan IM, Windle B. Analysis of The Cancer Genome Atlas sequencing data reveals novel properties of the human papillomavirus 16 genome in head and neck squamous cell carcinoma. Oncotarget. 2017;8(11):17684-99.

90. Mork J, Lie AK, Glattre E, Hallmans G, Jellum E, Koskela P, et al. Human papillomavirus infection as a risk factor for squamous-cell carcinoma of the head and neck. N Engl J Med. 2001;344(15):1125-31.

91. Gillison ML, Koch WM, Capone RB, Spafford M, Westra WH, Wu L, et al. Evidence for a causal association between human papillomavirus and a subset of head and neck cancers. J Natl Cancer Inst. 2000;92(9):709-20.

92. Halec G, Alemany L, Quiros B, Clavero O, Hofler D, Alejo M, et al. Biological relevance of human papillomaviruses in vulvar cancer. Mod Pathol. 2017;30(4):549-62.

93. De Vuyst H, Clifford GM, Nascimento MC, Madeleine MM, Franceschi S. Prevalence and type distribution of human papillomavirus in carcinoma and intraepithelial neoplasia of the vulva, vagina and anus: a meta-analysis. Int J Cancer. 2009;124(7):1626-36.

94. Joura EA, Leodolter S, Hernandez-Avila M, Wheeler CM, Perez G, Koutsky LA, et al. Efficacy of a quadrivalent prophylactic human papillomavirus (types 6, 11, 16, and 18) L1 virus-like-particle vaccine against high-grade vulval and vaginal lesions: a combined analysis of three randomised clinical trials. Lancet. 2007;369(9574):1693-702.

95. Evans MW, Sung AD, Gojo I, Tidwell M, Greer J, Levis M, et al. Risk assessment in human immunodeficiency virus-associated acute myeloid leukemia. Leuk Lymphoma. 2012;53(4):660-4.

96. Aboulafia DM, Meneses M, Ginsberg S, Siegel MS, Howard WW, Dezube BJ. Acute myeloid leukemia in patients infected with HIV-1. AIDS. 2002;16(6):865-76.

97. Sutton L, Guenel P, Tanguy ML, Rio B, Dhedin N, Casassus P, et al. Acute myeloid leukaemia in human immunodeficiency virus-infected adults: epidemiology, treatment feasibility and outcome. Br J Haematol. 2001;112(4):900-8.

98. Maiman M, Fruchter RG, Clark M, Arrastia CD, Matthews R, Gates EJ. Cervical cancer as an AIDS-defining illness. Obstet Gynecol. 1997;89(1):76-80.

99. Goedert JJ, Cote TR, Virgo P, Scoppa SM, Kingma DW, Gail MH, et al. Spectrum of AIDS-associated malignant disorders. Lancet. 1998;351(9119):1833-9.

100. Monforte A, Abrams D, Pradier C, Weber R, Reiss P, Bonnet F, et al. HIV-induced immunodeficiency and mortality from AIDS-defining and non-AIDS-defining malignancies. AIDS. 2008;22(16):2143-53.

101. Shiels MS, Pfeiffer RM, Hall HI, Li J, Goedert JJ, Morton LM, et al. Proportions of Kaposi sarcoma, selected non-Hodgkin lymphomas, and cervical cancer in the United States occurring in persons with AIDS, 1980-2007. JAMA. 2011;305(14):1450-9.

102. Frisch M, Biggar RJ, Engels EA, Goedert JJ, Group AI-CMRS. Association of cancer with AIDS-related immunosuppression in adults. JAMA. 2001;285(13):1736-45.

103. Grulich AE, van Leeuwen MT, Falster MO, Vajdic CM. Incidence of cancers in people with HIV/AIDS compared with immunosuppressed transplant recipients: a meta-analysis. Lancet. 2007;370(9581):59-67.

104. Yunis NA, Stone VE. Cardiac manifestations of HIV/AIDS: a review of disease spectrum and clinical management. J Acquir Immune Defic Syndr Hum Retrovirol. 1998;18(2):145-54.

105. Pugliese A, Isnardi D, Saini A, Scarabelli T, Raddino R, Torre D. Impact of highly active antiretroviral therapy in HIV-positive patients with cardiac involvement. J Infect. 2000;40(3):282-4.

106. Barbaro G. Cardiovascular manifestations of HIV infection. Circulation. 2002;106(11):1420-5.

107. Villa A, Foresti V, Confalonieri F. Autonomic neuropathy and prolongation of QT interval in human immunodeficiency virus infection. Clin Auton Res. 1995;5(1):48-52.

108. Kocheril AG, Bokhari SA, Batsford WP, Sinusas AJ. Long QTc and torsades de pointes in human immunodeficiency virus disease. Pacing Clin Electrophysiol. 1997;20(11):2810-6.

109. Sani MU, Okeahialam BN. QTc interval prolongation in patients with HIV and AIDS. J Natl Med Assoc. 2005;97(12):1657-61.

110. Katsarou O, Terpos E, Patsouris E, Peristeris P, Viniou N, Kapsimali V, et al. Myelodysplastic features in patients with long-term HIV infection and haemophilia. Haemophilia. 2001;7(1):47-52.

111. Takahashi K, Yabe M, Shapira I, Pierce S, Garcia-Manero G, Varma M. Clinical and cytogenetic characteristics of myelodysplastic syndrome in patients with HIV infection. Leuk Res. 2012;36(11):1376-9.

112. Dalakas MC, Pezeshkpour GH, Flaherty M. Progressive nemaline (rod) myopathy associated with HIV infection. N Engl J Med. 1987;317(25):1602-3.

113. Simpson DM, Bender AN. Human immunodeficiency virus-associated myopathy: analysis of 11 patients. Ann Neurol. 1988;24(1):79-84.

114. Feinberg DM, Spiro AJ, Weidenheim KM. Distinct light microscopic changes in human immunodeficiency virus-associated nemaline myopathy. Neurology. 1998;50(2):529-31.

115. Mirsattari SM, Power C, Nath A. Parkinsonism with HIV infection. Mov Disord. 1998;13(4):684-9.

116. Hersh BP, Rajendran PR, Battinelli D. Parkinsonism as the presenting manifestation of HIV infection: improvement on HAART. Neurology. 2001;56(2):278-9.

117. Koutsilieri E, Sopper S, Scheller C, ter Meulen V, Riederer P. Parkinsonism in HIV dementia. J Neural Transm (Vienna). 2002;109(5-6):767-75.

118. Almodovar S, Cicalini S, Petrosillo N, Flores SC. Pulmonary hypertension associated with HIV infection: pulmonary vascular disease: the global perspective. Chest. 2010;137(6 Suppl):6S-12S.

119. Speich R, Jenni R, Opravil M, Pfab M, Russi EW. Primary pulmonary hypertension in HIV infection. Chest. 1991;100(5):1268-71.

120. Mehta NJ, Khan IA, Mehta RN, Sepkowitz DA. HIV-Related pulmonary hypertension: analytic review of 131 cases. Chest. 2000;118(4):1133-41.

121. Opravil M, Pechere M, Speich R, Joller-Jemelka HI, Jenni R, Russi EW, et al. HIV-associated primary pulmonary hypertension. A case control study. Swiss HIV Cohort Study. Am J Respir Crit Care Med. 1997;155(3):990-5.
